# Function and Development of Deep-sea Mussel Bacteriocytes Revealed by snRNA-seq and Spatial Transcriptomics

**DOI:** 10.1093/gpbjnl/qzaf109

**Published:** 2025-11-25

**Authors:** Hao Chen (陈浩), Mengna Li (李梦娜), Zhaoshan Zhong (钟兆山), Inge Seim, Minxiao Wang (王敏晓), Chao Lian (连超), Lianhong Zhuo (卓联鸿), Xinjiang Wan (宛新江), Hao Wang (王昊), Guanghui Han (韩广辉), Li Zhou (周丽), Huan Zhang (张峘), Lei Cao (曹磊), Chaolun Li (李超伦)

**Affiliations:** Center of Deep Sea Research, Laboratory of Marine Ecology and Environmental Sciences, Institute of Oceanology, Chinese Academy of Sciences, Qingdao 266071, China; Center of Deep Sea Research, Laboratory of Marine Ecology and Environmental Sciences, Institute of Oceanology, Chinese Academy of Sciences, Qingdao 266071, China; National Deep Sea Center, Qingdao 266071, China; Center of Deep Sea Research, Laboratory of Marine Ecology and Environmental Sciences, Institute of Oceanology, Chinese Academy of Sciences, Qingdao 266071, China; Marine Mammal and Marine Bioacoustics Laboratory, Institute of Deep-sea Science and Engineering, Chinese Academy of Sciences, Sanya 572000, China; Center of Deep Sea Research, Laboratory of Marine Ecology and Environmental Sciences, Institute of Oceanology, Chinese Academy of Sciences, Qingdao 266071, China; Center of Deep Sea Research, Laboratory of Marine Ecology and Environmental Sciences, Institute of Oceanology, Chinese Academy of Sciences, Qingdao 266071, China; Center of Deep Sea Research, Laboratory of Marine Ecology and Environmental Sciences, Institute of Oceanology, Chinese Academy of Sciences, Qingdao 266071, China; Center of Deep Sea Research, Laboratory of Marine Ecology and Environmental Sciences, Institute of Oceanology, Chinese Academy of Sciences, Qingdao 266071, China; Center of Deep Sea Research, Laboratory of Marine Ecology and Environmental Sciences, Institute of Oceanology, Chinese Academy of Sciences, Qingdao 266071, China; Center of Deep Sea Research, Laboratory of Marine Ecology and Environmental Sciences, Institute of Oceanology, Chinese Academy of Sciences, Qingdao 266071, China; Center of Deep Sea Research, Laboratory of Marine Ecology and Environmental Sciences, Institute of Oceanology, Chinese Academy of Sciences, Qingdao 266071, China; Center of Deep Sea Research, Laboratory of Marine Ecology and Environmental Sciences, Institute of Oceanology, Chinese Academy of Sciences, Qingdao 266071, China; Center of Deep Sea Research, Laboratory of Marine Ecology and Environmental Sciences, Institute of Oceanology, Chinese Academy of Sciences, Qingdao 266071, China; South China Sea Institute of Oceanology, Chinese Academy of Sciences, Guangzhou 510301, China; Laoshan Laboratory, Qingdao 266071, China

**Keywords:** Chemosymbiosis, Host–symbiont interaction, Metabolic interaction, Immune regulation, Development plasticity

## Abstract

Deep-sea chemosynthetic ecosystems are among the most unusual ecosystems on Earth, where most megafauna form close symbiotic associations with chemosynthetic microbes to obtain nutrition and shelter from the toxic environment. Despite the diverse forms of symbiotic organs in these deep-sea holobionts, the function and development of bacteriocytes, the host cells harboring symbionts, are still largely uncharacterized. Here, we conducted an *in situ* decolonization assay and state-of-the-art single-nucleus and spatial transcriptomic analyses to reveal the function and development of deep-sea mussel bacteriocytes. Bacteriocytes appear to optimize immune processes to facilitate the recognition, engulfment, and elimination of endosymbionts. They also interact directly with endosymbionts in carbohydrate and ammonia metabolism by exchanging metabolic intermediates via transporters such as SLC37A2 and RHBG-A. Bacteriocytes arise from three different proliferation cell types, and their successive development trajectories were delineated using multi-omics data and 3D reconstruction analyses. The molecular functions and developmental processes of bacteriocytes are guided by the same set of molluscan-conserved transcription factors and may be influenced by endosymbionts through sterol metabolism. The coordination in the functions and development of bacteriocytes, and between the host and symbionts, highlights the phenotypic plasticity of symbiotic cells, and underpins host–symbiont interdependence in adaptation to the deep sea.

## Graphical abstract

**Figure:**
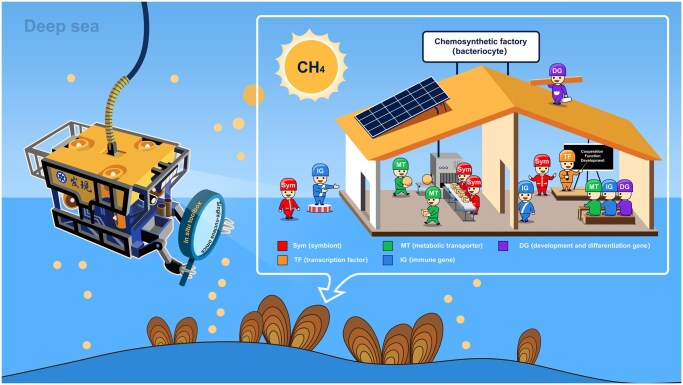

## Introduction

Symbiosis is a major driver of the acquisition of novel adaptive traits, expansion of ecological ranges, and shaping of biodiversity and evolution in eukaryotes [1]. To establish symbiotic associations, most animal hosts have evolved highly complex symbiotic cells and organs (*e.g.*, the aphid bacteriocytes, the bobtail squid light organ, and the tubeworm trophosome) that enable mutualistic relationships. Understanding the functions and development of symbiotic cells and organs is necessary to comprehend the formation and evolution of symbiosis [2]. With a growing number of studies, the functions and symbiotic interactions within symbiotic cells and organs are becoming clear in some model holobionts [3–5]. However, characterizing the function and development of non-model holobiont symbiotic cells and organs is challenging [6]. Furthermore, whether symbionts influence these functions and developmental programs, and by what mechanisms, remains debated and may vary greatly across species [7–9].

Since their discovery in 1977, chemosynthetic ecosystems in deep-sea cold seeps and hydrothermal vents have attracted considerable attention [10]. Notably, most endemic invertebrates in these ecosystems have evolved close symbiotic associations with chemosynthetic bacteria to obtain nutrients [11]. Mollusks, especially bivalves from the Mytilidae, Vesicomyidae, Solemyidae, Thyasiridae, and Lucinidae families, are particularly interesting because their chemosymbioses vary with the location of symbionts (extracellular or intracellular), type of symbiont (methanotroph, thiotroph, or both), and mode of transmission (vertical or horizontal) [12]. Moreover, whereas some mollusks develop bacteriocytes in their gills to host chemosynthetic endosymbionts, others may lose their symbionts in certain environments and undergo a reversal toward a non-symbiotic state, akin to their non-symbiotic shallow water counterparts [13]. For these reasons, mollusks are considered promising models for investigating chemosymbiosis — particularly the function and development of symbiotic cells and organs.

Among deep-sea mollusks, Bathymodiolinae mussels are known to harbor endosymbiotic methanotrophs and/or thiotrophs in their specialized gill epithelial cells (bacteriocytes) [14,15]. Adult deep-sea mussels contain 100 million to 1 billion bacteriocytes, each harboring hundreds of methanotrophic or thousands of thiotrophic endosymbionts [16]. Fine-scale metagenomic and meta-transcriptomic sequencing has revealed within-species diversity of symbiotic bacteria and functional differences among different symbiont strains [17,18]. Pathways and genes related to endocytosis, lysosomes, and central carbon metabolism have also been implicated by bulk RNA sequencing (RNA-seq) or proteomics of mussel gills, highlighting the potential metabolic and immune interactions between mussels and endosymbionts [19–21]. Additionally, new bacteriocytes can arise from proliferation cells adjacent to growth zones, outlining their developmental trajectory in the gill [22]. Interestingly, the structure of newly formed bacteriocytes changes drastically after symbiont infection, suggesting a potential role for symbionts in bacteriocyte function and development [22,23]. Although mussel bacteriocytes have been isolated in some studies, their precise functions and developmental processes remain insufficiently characterized due to the extensive heterogeneity of gill tissue and limitations in the isolation and *in vitro* culture of bacteriocytes and endosymbionts [24]. Recent advances in single-cell and spatial transcriptomics have allowed these issues to be addressed in non-model organisms without defined host cell lineages and culturable symbionts [25–27]. Recently, we constructed a comprehensive cell atlas of the gill of the methanotrophic deep-sea mussel *Gigantidas platifrons* using single-nucleus RNA sequencing (snRNA-seq) on the BD Rhapsody platform and characterized the expression pattern of mussel bacteriocytes [28]. While our data highlighted the unique functions of mussel bacteriocytes, how proliferation cells differentiate into bacteriocytes and whether a coordinated network controls their functions and development remain to be further addressed. Using high-resolution single-nucleus and spatial transcriptomic analyses, we conducted a comprehensive analysis, including a phagocytosis assay, 5-ethynyl-2′-deoxyuridine (EdU) labeling, and 3D electron microscopy to characterize the molecular functions and developmental trajectory of bacteriocytes. We also employed an *in situ* decolonization assay to compare our data with decolonized mussels and reveal the regulatory network governing the functions and development of symbiotic cells, as well as the potential influence of symbionts on this network.

## Results

### Gill cell lineage landscape

**Figure 1:**
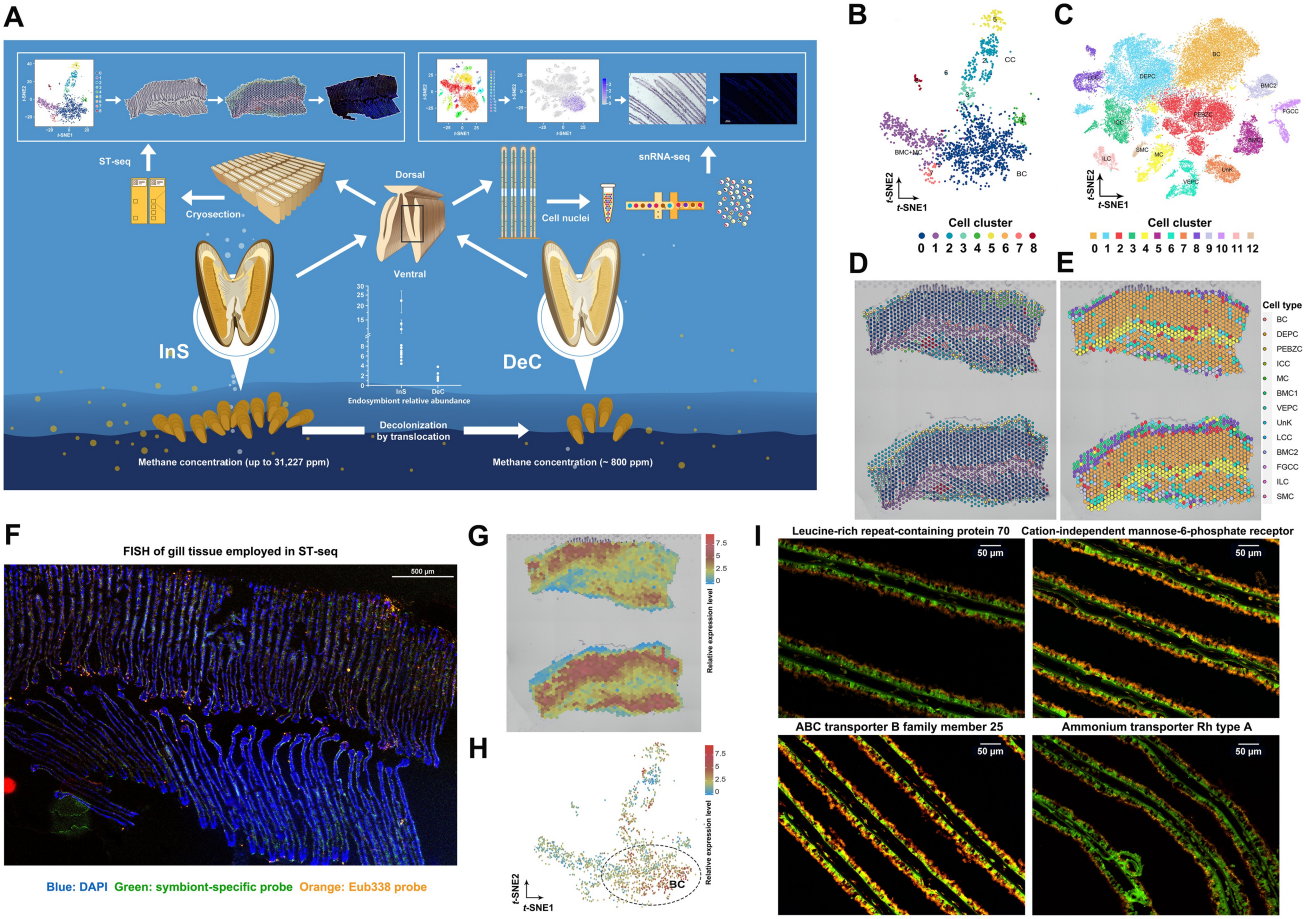
Spatially resolved single-cell transcriptomic atlas of deep-sea mussel gill **A**. Experimental workflow and analysis of snRNA-seq and ST-seq data of gill tissue in fully symbiotic (InS group) and partially decolonized (DeC group) deep-sea mussels. A marked decrease in the endosymbiont was observed after the *in situ* translocation assay, as calculated by comparing the relative DNA content of the symbiont methane monooxygenase gene with the internal control host β-actin gene by qRT-PCR (*n* = 14 for the InS group, *n* = 5 for the DeC group). **B**. *t*SNE projection of the spatial transcriptome clustered by gene expression in the InS group, with color assigned by cell type. **C**. Integrated *t*SNE projection of single-nucleus transcriptome clustered by gene expression in the InS (*n* = 3) and DeC (*n* = 1) group, with color assigned by cell type. **D**. Projection of cell clusters onto spatial transcriptome barcoded spots in the InS group. Two successive sections were used in the same capture region of the spatial transcriptome as technique replicates. **E**. Spatial transcriptome barcoded spots labeled using snRNA-seq cell type with maximum prediction score. **F**. FISH of endosymbionts with successive gill sections for ST-seq (the InS group). **G**. Expression patterns of bacteriocyte marker genes identified by snRNA-seq are depicted in the spatial transcriptome data. The relative levels, indicated with different colors, of the 219 shared marker genes identified in both snRNA-seq and ST-seq data were projected onto the barcoded spots. **H**. The relative levels of the 219 shared marker genes identified in both snRNA-seq and ST-seq data were projected onto the *t*SNE plot of the spatial transcriptome. **I**. Double-label FISH of selected marker genes (fluorescein-labeled gene-specific probe, green) from snRNA-seq and ST-seq data and symbionts (DIG-labeled Eub338 eubacteria probe, orange) was conducted to verify the bacteriocyte cluster. snRNA-seq, single-nucleus RNA sequencing; ST-seq, spatial transcriptomics sequencing; FISH, fluorescent *in situ* hybridization; *t*SNE, *t*-Distributed Stochastic Neighbor Embedding; DIG, digitoxin; BC, bacteriocyte; DEPC, dorsal end proliferation cell; PEBZC, posterior end budding zone cell; VEPC, ventral end proliferation cell; ICC, intercalary cell; LCC, lateral ciliary cell; FGCC, food grove ciliary cell; SMC, smooth muscle cell; BMC1, basal membrane cell 1; BMC2, basal membrane cell 2; MC, mucus cell; ILC, inter lamina cell; UnK, unknown cell; CC, ciliary cell.

To fully capture the expression patterns of key cell lineages, such as bacteriocytes and proliferation cells, we performed snRNA-seq on the 10x Genomics Chromium platform and spatial transcriptomics sequencing (ST-seq) on the 10x Genomics Visium platform using two groups of deep-sea mussels (**Figure 1**A). Mussels collected from the seepage region, where methane concentration is up to 31,227 ppm (*n* = 14), were used to represent mussels in a fully symbiotic state (designated as the InS group). Mussels that were transplanted *in situ* to a low methane geographic region for 604 days, with a methane concentration of approximately 800 ppm (39-fold lower than the seepage region) and an average 76.5% decrease in endosymbionts (calculated by quantitative real-time PCR of the methane monooxygenase gene in comparison with the relative DNA content) were used to represent partially decolonized mussels (designated as the DeC group; *n* = 5; Figure 1A, Figure S1A and B). Four mussels from the InS (three mussels) and DeC (one mussel) group with similar body size were randomly selected and subjected to snRNA-seq; two of these mussels (one mussel from each group) were also subjected to ST-seq. To include more possible cell types, the dorsal-middle region of the gill, which contains both descending and ascending gill filaments and is adjacent to the posterior end of the mussel, was employed in snRNA-seq. The cross-sectioned middle region of the gill, viewed dorsally, was used for ST-seq (Figure 1A).

Principal coordinate analysis (PCoA) of the meta-transcriptomic data for the two groups showed strong variation among individuals (Figure S1C and D). Approximately 57,776 cells (average 32,458 reads per cell) and over 3600 barcoded spots (average 172,705 reads per spot) were obtained for the snRNA-seq samples (four in total) and ST-seq samples (two in total), respectively (Table S1). Transcript coverage profiling showed that the sequencing depth of all samples was saturated on both sequencing platforms (Figure S1E–H), which was critical for subsequent analyses.

As expected, given our sampling strategy, more cell clusters (9 out of 13) were observed in both sample groups (InS and DeC) in the snRNA-seq dataset than in the ST-seq dataset (Figure 1B and C, Figure S2A). Among the 13 snRNA-seq cell clusters, 12 shared marker genes were reported in our previous study [28]. By comparing the shared marker genes with those from our previous study, we successfully identified bacteriocytes (cluster 0), three types of proliferation cells [dorsal end proliferation cells (DEPCs, cluster 1), posterior end budding zone cells (PEBZCs, cluster 2) and ventral end proliferation cells (VEPCs, cluster 6)], four types of ciliary and smooth muscle cells [intercalary cells (ICCs, cluster 3), lateral ciliary cells (LCCs, cluster 8), food grove ciliary cells (FGCCs, cluster 10), and smooth muscle cells (SMCs, cluster 12)], and four types of supportive cells [basal membrane cell 1 (BMC1, cluster 5), BMC2 (cluster 9), mucus cell (MC, cluster 4), and inter lamina cells (ILC, cluster 11)]. In addition, the median number of identified genes per cell and the total number of candidate marker genes both improved substantially in comparison with our previous study — where only 631 identified genes per cell per sample and 92 marker genes for bacteriocytes with at least 1.28-fold upregulated expression levels and *P* < 0.01 in the target cell cluster compared to the rest clusters were reported and with scRNA-seq of other deep-sea invertebrates [28,29]. In particular, the median number of identified genes per cell reached up to 900 and 2600 genes per cell/spot for snRNA-seq and ST-seq of the InS sample, respectively (Table S1); a total of 321, 81, 88, 173, 119, 147, 264, 113, 155, 177, 188, and 57 genes were identified as the marker genes of bacteriocyte, DEPC, PEBZC, VEPC, ICC, LCC, FGCC, SMC, BMC1, BMC2, ILC, and MC, respectively (Table S2).

To further verify the 13 identified cell types, particularly bacteriocytes, we combined the snRNA-seq and ST-seq data from the InS group (mussels in the fully symbiotic state) using Seurat v3.2’s anchor-based integration and projected the identified cell clusters from the snRNA-seq dataset onto the ST-seq data of the gill tissue (Figure 1D and E). While the fluorescence *in situ* hybridization (FISH) assay for eubacteria and methane oxidation symbionts confirmed that the cells of cluster 0 in the ST-seq data were bacteriocytes (Figure 1F), we further noticed that the majority of the annotated bacteriocyte marker genes from snRNA-seq data (219 out of 321) were also abundantly expressed in bacteriocytes from the ST-seq data (annotated as marker genes; Figure 1G and H; Table S2). Besides, the shared marker genes were found mainly expressed by bacteriocytes in the middle part of the gill filament, rather than by cells in the abfrontal region of the gill, where immature bacteriocytes are located (Figure 1G). Functional enrichment analysis based on Gene Ontology (GO) and double-label FISH of marker genes showed that bacteriocytes largely express genes involved in metabolic processes (*e.g.*, biosynthesis and transport of carbohydrates, lipids, amino acids, and vitamins) and symbiosis-related immune responses (including GO:0002376, GO:0009617, GO:0044111, and GO:0044403) (Figure S2B). Additionally, we noticed that 116 out of 321 marker genes were consistently expressed by bacteriocytes in the InS and DeC groups regardless of fluctuations in symbiont abundance (Table S3). These included the cation-independent mannose-6-phosphate receptor (Figure 1I), ABC transporter B family member 25 (Figure 1I, Figure S2C), leucine-rich repeat-containing protein 70 (Figure 1I), excitatory amino acid transporter 1, monocarboxylate transporter 13, cathepsin L (*ctsl*), and myoneurin (*mynn*) (Table S3). Of the remaining 205 marker genes, the majority (174) were downregulated in the DeC group in response to symbiont decolonization (Table S3). These included genes for the solute carrier family 40 member 1, ammonium transporter Rh type A (*rhbg-a*) (Figure 1I, Figure S2C), solute carrier family 26 member 10, 24-hydroxycholesterol 7 alpha-hydroxylase (*cyp39a1*, EC1.14.14.26), sugar phosphate exchanger 2 (*slc37a2*), zinc finger protein 271 (*znf271*), histone-lysine *N*-methyltransferase *ezh2,* and ETS-related transcription factor (*elf-3*) (Table S3).

### Symbiosis-related immune processes in bacteriocytes

**Figure 2:**
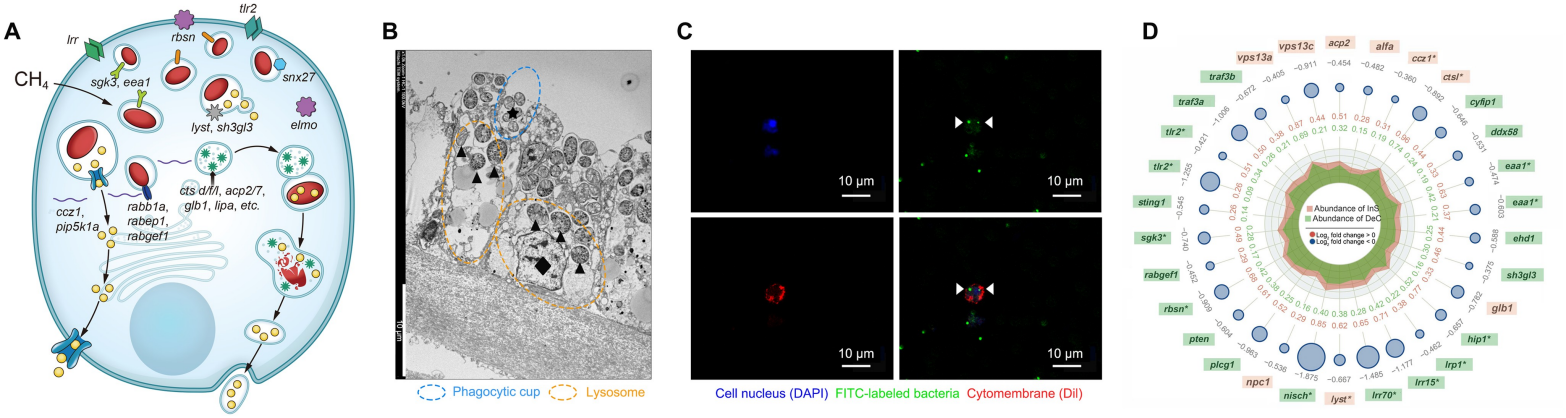
Immune genes and processes involved in symbiosis **A**. Schematic representation of immune-related marker genes of bacteriocytes. **B**. Endocytosis and lysosome-mediated digestion by bacteriocytes. Engulfment of symbiont-like bacteria (labeled with a star, in the phagocytic cup) by bacteriocytes was observed using TEM analysis. Some symbiont-like bacteria were also found to be digested inside lysosomes near the cell nucleus (labeled with a rhombus). **C**. Phagocytosis assay of mussel bacteriocytes with FITC-labeled bacteria. Phagocytosis assay by bacteriocytes conducted *in vitro* with FITC-labeled *Vibrio alginolyticus* (in green, indicated by a triangle) also confirmed that the bacteriocytes were able to engulf bacteria (cell nucleus stained with DAPI, cytomembrane stained with Dil). **D**. Expression alternations of endocytosis- and lysosome-related genes of bacteriocytes (including marker genes marked with “*” and non-marker genes) after symbiont decolonization. Almost all endocytosis (gene names labeled in green color) and lysosome (gene names labeled in pink color) related genes of bacteriocytes were downregulated in the DeC group in comparison with the InS group. The size of blue-colored apical circles represents the absolute value of log_2_ fold changes. The green and red-colored inner diagram represents the abundance of a given gene in the DeC and the InS group, respectively. TEM, transmission electron microscope; FITC, fluorescein isothiocyanate. *lrr*, leucine-rich repeat-containing protein; *tlr2*, Toll-like receptor 2; *sgk3*, serine/threonine-protein kinase 3; rbsn, rabenosyn-5; *eea1*, early endosome antigen 1; *snx27*, sorting nexin 27; *elmo*, engulfment and cell motility protein; *lyst*, lysosomal-trafficking regulator; *ccz1*, vacuolar fusion protein CCZ1; *pip5k1a*, phosphatidylinositol 4-phosphate 5-kinase type-1 alpha; *rabb1a*, Ras-related protein RABB1a; *rabep1*, Rab GTPase-binding effector protein 1; *rabgef1*, Rab5 GDP/GTP exchange factor; *alfa*, alpha-L-fucosidase; *acp*, lysosomal acid phosphatase; *sh3gl3*, endophilin-A3; *glb1*, beta-galactosidase; *lipa*, lysosomal acid lipase/cholesteryl ester hydrolase; *ddx58*, antiviral innate immune response receptor RIG-I; *ehd1*, EH domain-containing protein 1; *hip1*, huntingtin-interacting protein 1; *lrp1*, low-density lipoprotein receptor-related protein 1; *nisch*, Nischarin; *npc1*, NPC intracellular cholesterol transporter 1; *plcg1*, 1-phosphatidylinositol 4,5-bisphosphate phosphodiesterase gamma-1; *pten*, phosphatidylinositol 3,4,5-trisphosphate 3-phosphatase and dual-specificity protein phosphatase PTEN; *sting*, stimulator of interferon genes protein; *traf*, TNF receptor-associated factor; *vps*, vacuolar protein sorting-associated protein.

How invertebrate hosts, armed with innate immunity alone, forged a symbiotic association with specific chemosynthetic bacteria remains intriguing. We screened for genes and pathways that may participate in the establishment and maintenance of symbiosis in bacteriocyte lineages. Although pattern recognition receptors (PRRs), such as Toll-like receptors (TLRs) and peptidoglycan recognition proteins (PGRPs), could play vital roles in the recognition of symbionts in other holobionts [30], we found that only a few canonical PRR genes were highly expressed in bacteriocytes of mussels in the fully-symbiotic state (the InS group) or decolonized mussels (the DeC group), while numerous PRRs showed relatively low levels of expression or were not characterized. For example, only one Toll-like receptor gene, *tlr2*, was identified as a marker for bacteriocytes (**Figure 2**A; Table S2), while the expression levels of two PRRs, *tlr2* and *tlr6*, decreased in bacteriocytes after decolonization (Table S3). In addition, snRNA-seq failed to characterize 55 out of 146 *tlr* genes. In comparison, four leucine-rich repeat-containing proteins (LRRs) were found to be abundantly expressed in bacteriocytes, which were suggested to be potential receptors for endosymbionts in previous studies [31].

Meanwhile, we detected abundant expression of endocytosis- and lysosome-related marker genes, such as *rabenosyn-5* (*rbsn*), early endosome antigen 1 (*eea1*), sorting nexin-27 (*snx27*), lysosome trafficking regulator (*lyst*), endophilin-A3 (*sh3gl3*), cathepsin D/F/L (*ctsd*/*f*/*l*), acid phosphatase type 2 (*acp2*) and acid phosphatase type 7 (*acp7*), in bacteriocytes from fully symbiotic mussels (Figure 2A; Table S2). The high expression level of these genes supported the transmission electron microscopy (TEM) analysis and phagocytosis assay findings that bacteriocytes uptake and digest bacteria (Figure 2B and C, Figure S2D and E; Video S1). In addition, although the endosymbionts are often found digested within lysosomes (Figure S2E), they are rarely observed to proliferate within bacteriocytes. For example, only 3 out of 408 symbionts per bacteriocyte were likely to proliferate within bacteriocytes, as evidenced by 3D electron microscopy analysis (Figure S2E; Video S2). Meanwhile, most endocytosis- and lysosome-related marker genes of bacteriocytes (as well as some non-marker genes) were downregulated as symbiont numbers decreased (Figure 2; Table S3).

### Metabolic interactions between the host and symbionts

**Figure 3:**
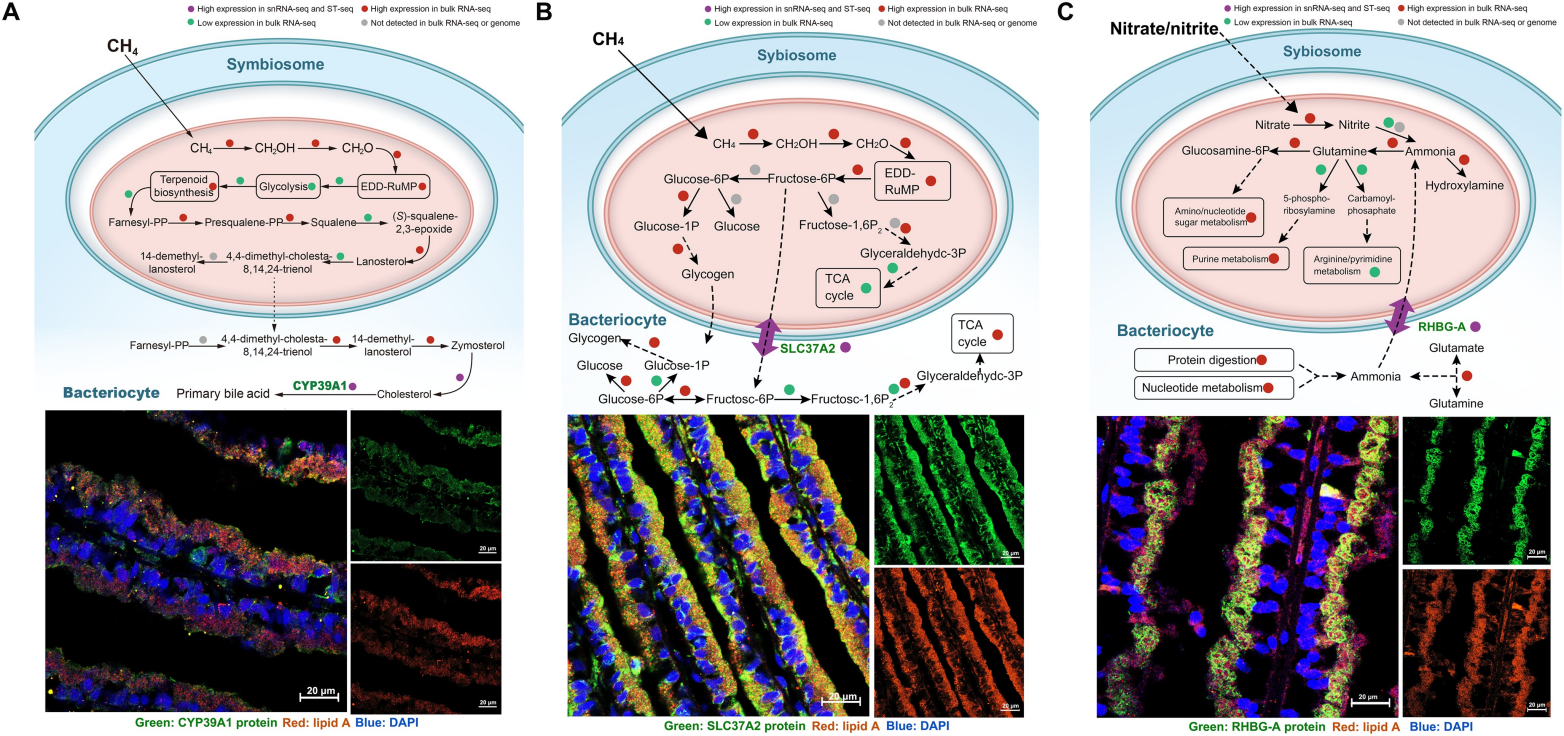
Metabolic interactions between mussel host and endosymbionts **A**. Interaction of sterol metabolism between the host and symbionts. The snRNA- and ST-seq data showed the abundant expression of *cyp39a1* in bacteriocytes. CYP39A1 plays a crucial role in host cholesterol metabolism, which relies heavily on sterol intermediates provided by symbionts, as evidenced by genomic and meta-transcriptome data. IF assays also show that most CYP39A1 proteins (in green) are distributed in the apical region of bacteriocyte, which is enriched with endosymbionts (indicated by lipid A signals, in red). **B**. Metabolic interaction of sugar phosphate between the host and symbionts. Although genomic and meta-transcriptome data showed abundant expression of glycogen synthesis genes in both host and symbiont, genes converting fructose-6P to glucose-6P or fructose-1,6P_2_ are not detected in the symbiont. Nonetheless, these genes are encoded and actively expressed in mussel gills. In addition, snRNA and ST-seq data showed an abundant expression of the *slc37a2* gene by bacteriocytes. Further IF assays showed the co-location of SLC37A2 proteins (in green) with endosymbionts (in red) inside bacteriocytes. **C**. Interactions of ammonia metabolism between the host and symbionts. Ammonia, a by-product of host metabolism, plays a crucial role in symbiont production of hydroxylamine and glutamine. The mussel host was found to be massively transcribing ammonium transporter genes (up to three different genes) in bacteriocytes, potentially allowing direct import of ammonia for the symbionts. In support of this, IF assays showed the co-location of RHBG-A proteins (in green) with endosymbionts (in red) inside bacteriocytes. IF, immunofluorescence; CYP39A1, 24-hydroxycholesterol 7 alpha-hydroxylase; SLC37A2, sugar phosphate exchange; RHBG-A, ammonium transporter Rh type A; EDD-RuMP, Entner-Doudoroff pathway and ribulose monophosphate cycle.

Deep-sea mussels obtain and transport nutrients derived from symbionts within their bacteriocytes. Functional enrichment analysis revealed that metabolic genes were more highly expressed in bacteriocytes (Figure S2B). These metabolic marker genes are associated with the biosynthesis and transport of carbohydrates, lipids, amino acids, and vitamins, implying metabolic interactions between deep-sea mussels and their symbionts. Among these, the marker gene *cyp39a1* (EC1.14.14.26) was highly expressed in bacteriocytes from fully symbiotic mussels (the InS group) based on both snRNA-seq and ST-seq data (**Figure 3**A). CYP39A1 is a key enzyme involved in the turnover of sterol intermediates, such as presqualene-PP, squalene, (*S*)-squalene-2,3-epoxide, and 4,4-dimethyl-cholesta-8,14,24-trienol [32]. The colocalization of CYP39A1 (EC1.14.14.26) with the endosymbiont was further confirmed by immunofluorescence (IF) of bacteriocytes (Figure 3A). We also noticed that the mussel genome lacked related enzymes (*e.g.*, EC2.5.1.21, EC1.14.1417, EC1.14.14154, and EC1.14.1536) required to synthesize the above sterol intermediates. Nevertheless, meta-transcriptome data showed that methanotrophic endosymbionts robustly expressed the genes encoding enzymes such as EC2.5.1.21 and EC1.14.14154 (the top 10% highly expressed genes in the InS group) in a complementary manner to the host’s own expression (Figure 3A, Figure S4A–D; Table S4), evidencing the metabolic dependency of mussel hosts on sterol-derived intermediates of symbionts [33].

Both the mussel host and endosymbionts highly expressed key gluconeogenesis and glycogen biosynthesis genes (*e.g.*, EC2.4.1.18, 2.4.1.21, 2.7.7.27, and 5.4.2.2), as demonstrated by meta-transcriptome data, with these genes being among the top 10% of highly expressed genes in the InS group (Figure 3B, Figures S5 and S6; Table S4), implying robust production of glycogen in mussels and endosymbionts. However, we observed that multiple enzymes in the gluconeogenesis pathway of the endosymbiont were either insufficiently expressed, such as EC5.3.1.9, or completely absent from the genome, including EC3.1.3.11, EC2.7.1.11, and EC4.1.2.13, which is likely to result in overproduction of fructose-6P and shortages of glucose-6P, fructose-1,6P_2_, glyceraldehyde-3P, and glucose and reduced glycogen production by the symbiont and host. Conversely, the mussel host expressed all these enzymes; moreover, a sugar phosphate exchanger gene *slc37a2* (responsible for transmembrane transport of fructose-6P, glucose-6P, fructose-1,6P_2_, and glyceraldehyde-3P) was found abundantly expressed by bacteriocytes, as shown by snRNA-seq and ST-seq of the InS group, and co-localized with endosymbionts (Figure 3B, Figure S6). The complementation in carbohydrate metabolism strongly suggests that symbionts can supply fructose-6P directly to the host in exchange for gluconeogenesis intermediates. Consistent with this observation, we also noticed that the endosymbionts were massively transcribing phosphate and carboxylate transporters (including the phosphate-binding protein PstS, maltose 6-phosphate phosphatase, phosphate permease, sodium-dependent dicarboxylate transporter SdcS, and bicarbonate transporter BicA; Table S4), which may facilitate the export of sugar phosphate compounds.

TEM images of bacteriocytes have shown that endosymbionts are contained in separate vacuoles designated as symbiosomes [34]. While the presence of symbiosomes creates a suitable micro-environment for endosymbionts, they also limit access to environmental nutrients such as ammonia. As a by-product of protein digestion and nucleotide metabolism, ammonia is harmful to mussel hosts’ cells and tissues but is necessary for endosymbionts. Specifically, we observed higher expression of ammonia consumption-related genes (top 10%) rather than ammonia production-related genes in endosymbionts (Figure 3C, Figure S7; Table S4). Meanwhile, our snRNA- and ST-seq data also revealed that up to three ammonium transporter genes were highly expressed in bacteriocytes (Figures 1G and 3C), which may facilitate the transport of ammonia to symbiosomes. In addition, we noted that the RHBG-A protein, one of the most abundantly expressed ammonium transporters in bacteriocytes, co-localized with endosymbionts (Figure 3C). At the same time, endosymbionts also abundantly transcribed ammonium transporter genes (such as the ammonia channel gene, top 10% expressed genes in the InS group; Table S4), collectively evidencing the direct flow of ammonia from host to symbiont.

We also found that the expression levels of genes involved in host–symbiont metabolic interactions (*i.e.*, *cyp39a1*, *rhbg-a*, and *slc37a2*) were markedly downregulated in bacteriocytes of the DeC group when the methane supply was limited and when symbiont abundance decreased (Figure S8). Taken together, these findings highlight the possibility that mussel hosts interact with symbionts dynamically based on the environment and symbiont abundance.

### Coordinated regulatory networks guide molecular functions in bacteriocytes

While the above results collectively suggest that the host optimizes its immune and metabolic processes to facilitate symbiosis, we questioned whether a coordinated regulatory network guides these processes. To construct the regulatory network, we conducted weighted gene co-expression network analysis (WGCNA) using snRNA-seq data from the fully-symbiotic (InS group) and decolonized mussels (DeC group). One WGCNA module, Mod05, contained 225 out of 321 marker genes for bacteriocytes (Figure S9A; Tables S5 and S6), suggesting that it represents the network regulating bacteriocyte function. Functional enrichment analysis demonstrated that these 225 genes are involved in the immune and metabolic processes of bacteriocytes (Figure S9B; Table S7).

**Figure 4:**
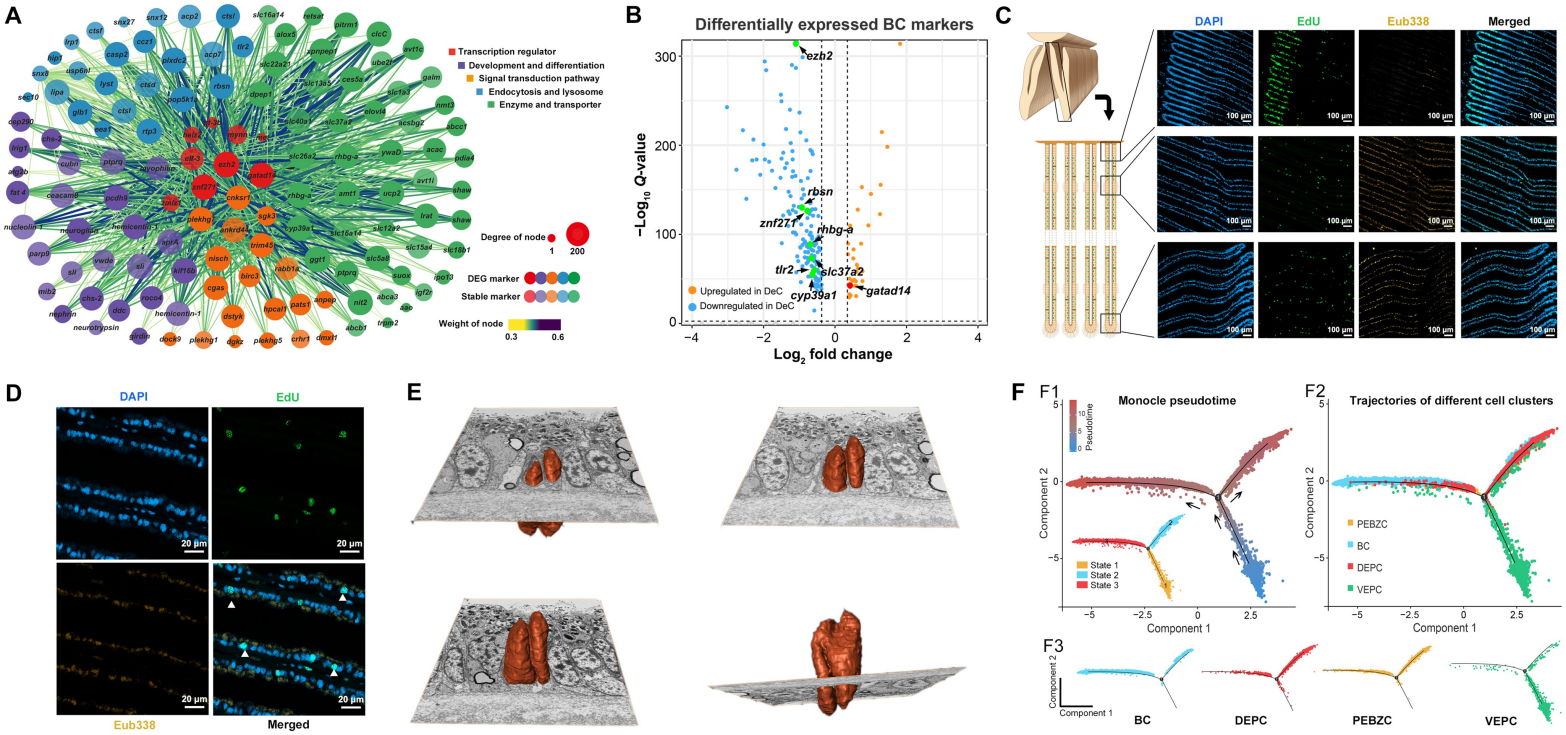
Development trajectory of deep-sea mussel bacteriocyte lineages **A**. A genetic regulatory network of bacteriocytes was constructed using WGCNA with snRNA-seq data. Marker genes with similar functions or involved in the same biological processes are labeled with the same color. The WGCNA weight between two given nodes is indicated by different edge colors, and the degree of a given node is indicated by the dot size. **B**. Expression patterns of bacteriocyte markers in decolonized mussels (DeC group) in comparison with fully symbiotic mussels (InS group). Dozens of marker genes were consistently expressed by bacteriocytes, regardless of variations in symbiont abundance, recognized as stable markers and displayed in translucent colors. The remaining markers, including some hub transcription factors of the genetic regulatory network, were found to be downregulated in the DeC group (in solid colors), accompanied by differential expression of 148 other element markers (the X-axis represents the log_2_-scaled ratio of fold changes between the DeC and InS groups, with dashed vertical lines at ± 0.36 denoting the fold change threshold, and the Y-axis represents the −log_10_  *Q*-value of the significant difference test, with a dashed horizontal line at 2 indicating the significance threshold). **C**. Distribution patterns of proliferation cells in the InS group as revealed by an EdU staining assay. Newly synthesized DNA was labeled with EdU signals (green) while cell nuclei were stained by DAPI (blue), and symbionts were labeled using a Cy3-labeled Eub338 probe (orange). **D**. Newly proliferated bacteriocytes observed by EdU assay and 3D electron microscopy. EdU signals indicating DNA replication are observed in the nucleus region of some bacteriocytes of the InS group (white triangle). **E**. Nuclear division in bacteriocytes could also be observed with 3D reconstruction of gill tissues (a total of 91 serial images with a thickness of 100 nm per section). **F**. Development trajectory of bacteriocyte lineages. Pseudo-time analysis in Monocle shows the successive development process from proliferation cells to bacteriocytes, which could be further divided into three distinct states (F1). Proliferation cells and ciliated cells are distributed mostly in states 1 and 2, while bacteriocytes are mostly distributed in states 2 and 3 (F2, 3). WGCNA, weighted gene co-expression network analysis; EdU, 5-ethynyl 2′-deoxyuridine; *znf271*, zinc finger protein 271; *elf*-3, ETS-related transcription factor 3; *ezh2*, histone-lysine *N*-methyltransferase 2; *gatad14*, GATA zinc finger domain-containing protein 14; met, hepatocyte growth factor receptor; *mynn*, zinc finger protein myoneurin; *zmiz1*, zinc finger MIZ domain-containing protein 1; *helz2*, 3′–5′ exoribonuclease HELZ2; *gt*-3b, trihelix transcription factor GT-3b. Abbreviations for the remaining genes in Figure 4A are listed in Table S6.

Next, we screened for highly expressed transcription factors and signal transducers within the regulatory network that may function as hub regulators of bacteriocyte function. Within the regulatory network of bacteriocytes, we identified nine transcription factors, including *znf271*, a zinc finger protein; *elf-3*, an ETS-related transcription factor; *ezh2*, a histone-lysine *N*-methyltransferase; *gatad14*, a GATA zinc finger domain-containing protein; *met*, the hepatocyte growth factor receptor; *mynn*, a zinc finger protein myoneurin; *zmiz1*, a zinc finger MIZ domain-containing protein; *helz2*, a helicase with a zinc finger domain; and *GT-3b*, a trihelix transcription factor (**Figure 4**A). These transcription factors were highly expressed in bacteriocytes and correlated with the expression of 225 out of 321 bacteriocyte marker genes, including the aforementioned metabolic genes (*cyp39a1*, *slc37a2*, and *rhbg-a*) and immune-related genes (*tlr2*, *rbsn*, *lyst*, and cathepsins) (Figure 4A). The expression of *ezh2*, *znf271*, and *gatad14* was also differentially regulated during decolonization, in conjunction with the differential expression of 148 other marker genes (Figure 4B). Moreover, strong interconnectivity (WGCNA weight > 0.3) was observed among these transcription factor genes (Figure 4A), indicating that they may regulate each other synergistically.

### Successive trajectory of the development of bacteriocyte lineages

Without culturable samples of post-larval and juvenile deep-sea mussels, the mechanisms underlying the development of bacteriocyte lineages remain elusive. As reported in our previous study [28], there are three types of proliferation cells in the gills of mussel hosts: PEBZCs, DEPCs, and VEPCs. By conducting an EdU-labeling assay, we confirmed widespread proliferative activity along the gill filaments, especially in the dorsal part of the gills, where DEPCs are located (Figure 4C, Figure S8D). Additionally, we observed proliferating signals in some bacteriocytes of adult mussels, as assessed using EdU-labeling and 3D electron microscopy (Figure 4D and E; Video S3). While robust proliferation signals confirmed the continuous birth of bacteriocytes in adult mussel gills, the identification of marker genes for bacteriocytes and proliferation cells provides a unique opportunity to advance our knowledge of bacteriocyte development. Using an expression atlas for proliferation cells and bacteriocyte lineages, we also characterized the bacteriocyte development trajectory with five different models: pseudo-time analysis by Monocle2 (Figure 4F), partition-based graph abstraction (PAGA) analysis, Slingshot, Cytotrace, and RNA velocity analysis (Figure S9C and D). The Monocle analysis showed that gill cells were distributed across three distinct pseudo-time states. Most proliferation cells, as the starting point of differentiation, were in states 1 and 2 (for example, 93.41% of VEPCs were in state 1, while 84.02% of DEPCs and 50.77% of PEBZCs were in state 2, respectively). Conversely, as differentiation endpoints, approximately 94.96% of bacteriocytes were in state 3 (Figure 4F). Similar results were also observed in the other analyses, where bacteriocytes were found in a more differentiated state than DEPCs, PEBZCs, and VEPCs (Figure S9C). These distinct pseudo-time trajectory states collectively confirm developmental heterogeneity within gill cells and position bacteriocytes as one of the most differentiated cells in mussel gills. Particularly, a succession of processes occurred during differentiation (cell type transitions from proliferation cells, especially from DEPCs, to bacteriocytes) and maturation (subtype transitions from state 2 to state 3) of bacteriocytes (Figure 4F, Figure S9C and D).

To further identify the cell differentiation processes in bacteriocyte lineages, we examined the genes abundantly expressed at different pseudo-time states (Table S8). We speculated that progenitor states would encode key marker genes (especially transcription factors) of descendant cells, and that the expression levels of these marker genes would increase as cells mature, showing maximal expression levels in the fully functional state. Our results indicate that bacteriocytes at state 3 are in a fully functional state, transcribing the majority of marker genes (including hub marker genes) (Table S8). Bacteriocytes in state 2 were functionally immature and showed moderate expression of marker genes. Notably, a large number of genes abundantly expressed at state 3 of proliferation cells (128 out of 625 genes in DEPCs, 121 out of 621 genes in PEBZCs, and 70 out of 101 genes in VEPCs) were bacteriocyte marker genes (Figure S9E). Meanwhile, 33 out of 81 DEPC marker genes were also highly expressed in state 2 PEBZCs (Table S8). These findings suggest that the state-3 proliferation cells may be bacteriocyte progenitors, while DEPCs may be descendants of PEBZCs. This conclusion is also supported by RNA velocity analysis and the Slingshot model, in which a positive velocity from PEBZCs and DEPCs to bacteriocytes was observed in the corresponding cells of adjacent regions (Figure S9C).

### Co-option of conserved transcription factors in bacteriocyte development

**Figure 5:**
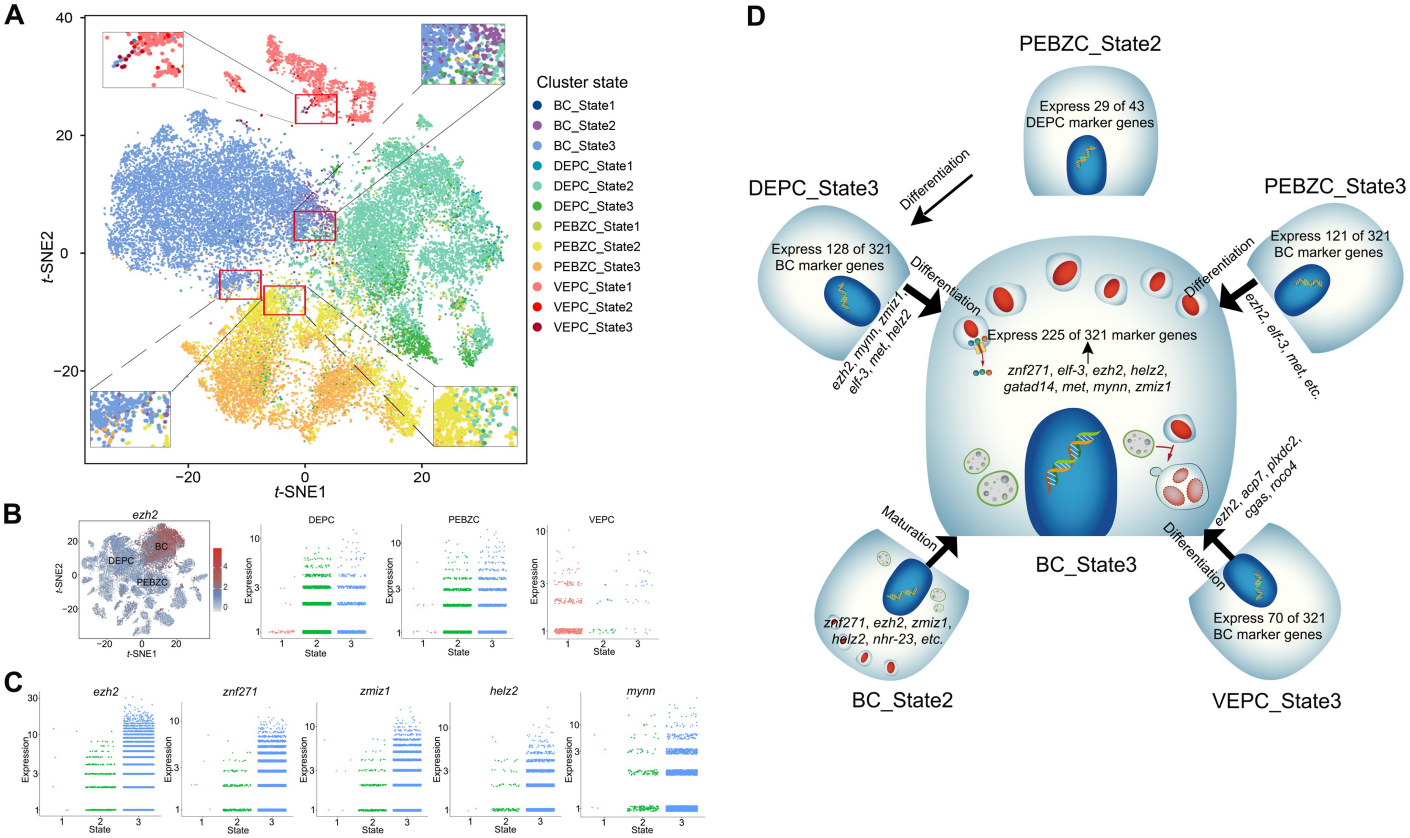
Co-option of conserved transcription factors in the function and development of bacteriocytes **A**. Proliferation cells and bacteriocytes in different pseudo-time states were projected on the *t*SNE plot of snRNA-seq data. Co-overlaps in the *t*SNE distribution of bacteriocytes and state 3 cells of proliferation cells (such as DEPC and PEBZC) and between DEPC and state 2 cells of PEBZC were observed (shown in magnified fields). **B**. Expression patterns of the hub transcription factor *ezh2* in color-coded *t*SNE plots (left, color indicating the relative expression level) and different states of proliferation cells (DEPC, PEBZC, and VEPC). **C**. Expression patterns of hub transcription factors in different states of bacteriocytes. **D**. Schematic diagram shows the function and development of bacteriocyte lineages guided by the co-option of conserved transcription factors identified from the genetic regulatory networks. Arrows represent the deduced developmental trajectory (including cell type transition and maturation) in bacteriocytes.

While the development of symbiotic cells and organs may be a highly organized process, the regulatory networks that guide it are difficult to identify in both model and non-model symbiotic associations. Using our bacteriocyte developmental trajectory, we explored the mechanisms underlying bacteriocyte differentiation and maturation, focusing on genes that are abundantly expressed in the progenitor state. By conducting *t*-Distributed Stochastic Neighbor Embedding (*t*SNE) analysis, we observed an overlap in the *t*SNE distributions between state 2 bacteriocytes and state 3 proliferation cells, and an overlap between state 2 DEPCs and state 2 PEBZCs (**Figure 5**A), confirming that state 3 DEPCs/PEBZCs are progenitors of bacteriocytes. Specifically, our results indicate that the mRNA transcripts of histone-lysine *N*-methyltransferase *ezh2*, a hub marker gene in the regulatory network of bacteriocytes and a canonical regulator of gene expression and cell differentiation, are abundantly expressed in state 3 of all proliferation cell types (Figure 5B; Table S8). Additionally, the expression of plexin domain-containing protein *plxdc2*, *acp7*, cyclic GMP-AMP synthase (*cgas*), and the serine/threonine-protein kinase *roco4*, which are bacteriocyte marker genes identified in the regulatory network and crucial regulators of cell differentiation and lysosomal function, was also highly upregulated in state 3 of all proliferation cells (Figure S9F; Table S8). In addition, the expression of five other hub transcription factors in the regulatory network of bacteriocytes robustly increased in state 3 DEPCs (*elf-3*, *mynn*, *helz2*, *zmiz1*, and *met*) and two in PEBZCs (*elf-3* and *met*) in comparison with the other cell states (Table S8), suggesting their participation in the transition of proliferation cells into bacteriocytes.

Similarly, a gradual increase in the expression levels of the four aforementioned hub genes (*znf271*, *ezh2*, *zmiz1*, and *helz2*) was also observed during further maturation (from state 2 to state 3) of bacteriocytes (Figure 5C; Table S8). Additionally, 13 transcription factors, which act as activators or repressors, were highly expressed in state 2 bacteriocytes (Figure S9F; Table S8). To address the potential influences of symbionts on the function and development of bacteriocytes, we further searched for sterol- and sugar phosphate-related genes that were differentially expressed in state 2 of bacteriocytes and after the decolonization assay, as sterols and sugar phosphates are two main nutrients derived from symbionts. Particularly, we observed that the nuclear hormone receptor family member *nhr*-*23* was abundantly expressed in state 2 (Figure S9F; Table S8). In addition, seven genes involved in the sterol-related signaling pathway (including *cyp39a1* and six nuclear receptor genes) were also differentially expressed in bacteriocytes of decolonized mussels (Table S3). Nuclear receptor genes, including *nhr-23*, are known to play a crucial role in the sterol-related signaling pathway by binding to steroidal ligands and regulating cell function and development.

While the aforementioned hub transcription factors and signaling transducers may be trigger molecules promoting bacteriocyte development, we asked whether they were evolutionarily novel genes that diverged from non-symbiotic ancestors or conserved across evolution. Our phylogenetic analysis of protein sequences obtained from homologues of these genes in Lophotrochozoa indicates that these genes are highly conserved in mollusks and are in accordance with the general host phylogeny (Figure S10). We propose that bacteriocyte development occurred through the co-option of conserved genes rather than the emergence of evolutionarily novel genes (Figure 5D).

## Discussion

In addition to representing an evolutionary novelty, symbiotic cells and organs provide a suitable niche for symbiosis and endow holobionts with unique adaptations to their surrounding environments [6]. In this study, we integrated data obtained from single-nucleus omics, spatial omics, phagocytosis assays, EdU labeling assays, and 3D electron microscopy to uncover the regulatory network that controls the function and development of bacteriocytes in deep-sea mussels. Our findings suggest that these processes are guided by an ancestral intrinsic toolkit through the co-option of conserved transcription factors and modulation by endosymbionts. Our results suggest how the two partners interact closely and cooperatively shape the function and development of symbiotic cells, which is crucial for understanding the evolution of chemosymbiosis and the adaptations of holobionts in habitats such as the deep sea [35,36].

### The sophisticated immune network controls the “ins” and “outs” of endosymbionts

The establishment and maintenance of endosymbiosis with exogenous bacteria are challenging for multicellular organisms because of the host immune system, which requires adjustments that entail engulfing and proliferating symbionts within the host cell [37]. Although it is well recognized that some PRRs have greatly expanded in the deep-sea mussel genome and could act as immune receptors for symbiont [19], only a few canonical PRRs were found to be abundantly expressed in bacteriocytes, either in our snRNA-seq data or ST-seq data. Instead, several *lrr* genes were found to be abundantly expressed in bacteriocytes, which have been suggested as potential intracellular receptors for methanotrophic endosymbionts in previous studies [31]. While the aforementioned canonical PRRs might participate in other processes, such as early symbiotic acquisition in juveniles and defense against pathogenic bacteria, our findings suggest that only a few PRRs, including some non-canonical ones, are needed to maintain symbiosis in normal bacteriocytes of adult mussels. Nevertheless, bacteriocytes can still phagocytize exogenous bacteria in a nonselective manner, even when fully colonized by endosymbionts, as observed in our phagocytosis assay and in some recent studies [38]. The phagocytic ability of gill cells may be conserved in most mollusks, including non-symbiotic ones [38–40], which directly facilitates the entry of endosymbionts into deep-sea mussels and may contribute significantly to the establishment of symbiosis in other mollusks. To facilitate phagocytosis and subsequent lysosome-mediated digestion of bacteria, a mass of phagosome- and lysosome-related genes was also found to be abundantly expressed by bacteriocytes. For example, *rbsn* and *eez1*, as two marker genes of bacteriocytes, could be recruited to early endosomes and therefore play a crucial role in the formation of the phagocytic cup; the *rab* family and *snx27* are essential for intracellular trafficking, especially between phagosomes and intracellular compartments [41]. Similarly, *lyst* and *sh3gl3* could play a necessary role in lysosome maturation, while *ctsd/f/l* and *acp2/7* are required for lysosome-mediated biodegradation. The massive expression of the aforementioned genes collectively underpins the unique function and cell structure of bacteriocytes, as observed by phagocytosis assay and TEM analysis. However, the phagocytosis of exogenous bacteria also raises questions about whether and how mussels discriminate between symbionts and non-symbionts, and between symbionts in good and bad states, within phagosomes or symbiosomes, since it is often observed that bacteriocytes digest some bacteria resembling symbionts via lysosomes. Recently, it has been reported that the digestion of symbionts is regulated by mTORC1, particularly under conditions of reduced nutrient supply (either due to endosymbiont death or methane removal) [42–44]. At the same time, we rarely observed proliferating endosymbionts or free symbionts engulfed in bacteriocytes of adult mussels, as evidenced by EdU labeling assay and 3D electron microscopy. We therefore speculated that lysosome-mediated symbiont digestion is a secondary option to obtain nutrition under normal circumstances, but an efficient way to control the symbiont population and obtain nutrition under abrupt stresses or other emergencies. In support of this speculation, genes involved in phagosome and lysosome maturation were found massively downregulated in bacteriocytes of decolonized mussels. Our findings also support that symbiotic associations in deep-sea mollusks are nutrition-driven and may involve metabolic interactions between mussel hosts and symbionts [11].

### The close interactions between the metabolism of mussels and endosymbionts

Since the first characterization of chemosymbiosis, it has long been debated how invertebrate hosts, including deep-sea mussels, acquire nutrients from and supply essential substances to the symbionts. Besides the lysosome-mediated digestion of symbionts, mussel hosts are suggested to acquire nutrients through the uptake of secreted symbiont metabolites [38]. However, identifying the exact metabolites and the mechanisms of their transport between symbionts and bacteriocytes has remained unclear and difficult to characterize [19,20,33,45,46]. Genomic information shows that methanotrophic endosymbionts can provide sterol intermediates to the mussel hosts. Geier et al. recently identified several specialized metabolites at the host-microbe interface using metaFISH and AP-MALDI-MSI analyses [46]. With the help of state-of-the-art single-nucleus and spatial sequencing, we here provide evidence that deep-sea mussels have significantly reshaped bacteriocyte metabolism to maximize symbiotic profits for both partners. For example, bacteriocytes encode dozens of genes involved in the biosynthesis and transport of carbohydrates, lipids, amino acids, and vitamins, which improve acquisition of nutrients from symbionts. Our findings also reveal that bacteriocytes express the sugar-phosphate exchanger gene *scl37a2*, which co-localizes with symbionts and may facilitate the retrieval of fructose-6P directly from methanotrophic symbionts. As a member of the SLC37 family, SLC37A2 is typically associated with the endoplasmic reticulum (ER) and functions as a Pi-linked antiporter for fructose-6P or glucose-6P [47]. While it is widely acknowledged that the ER plays a role in the formation and development of symbiosomes [48], our results provide a vivid example of how ER-associated proteins like SLC37A2 specifically contribute to symbiosome function. In bacteriocytes, fructose-6P is further converted into glucose-6P, fructose-1,6P_2_, and glyceraldehyde-3P, which could support the gluconeogenesis and the tricarboxylic acid (TCA) cycles in both mussel hosts and symbionts. In addition, we suggest that mussel hosts supply ammonia to symbionts directly via ammonium transporters *rhbg-a*. Although ammonium is a preferred nitrogen source for endosymbionts, it is toxic to animal hosts and must be excreted. While ammonium transporters typically belong to three superfamilies (Amt/Mep/Rh), the AMT superfamily is most commonly associated with ecto- and endomycorrhizal symbiosis in plants [49]. In contrast, this study provides the first evidence of how RHBG-A, a member of the Rh superfamily, facilitates ammonia transport in symbiotic relationships. Similarly, it has been shown that the thiotrophic symbionts of *Riftia* can release succinate directly to the host after CO_2_ fixation; the sea anemone *Aiptasia* can also provide ammonia directly to symbiotic algae and control their growth [50,51]. Moreover, a recent study on vestimentiferan tubeworms showed that thiotrophic symbionts massively transcribe ammonia transporter genes to absorb and detoxify ammonia from the host [29]. The direct transport of fructose-6P and ammonia in mussel holobionts, therefore, could provide a shortcut for both mussel hosts and methanotrophic symbionts to obtain essential nutrients, which may greatly increase the efficiency and profits of symbiosis to adapt to the harsh environment of the deep sea and serve as a supplementary way to control the symbiont population. In support of this speculation, it is well recognized that a single bacteriocyte can harbor up to thousands of methanotrophic symbionts, while the free-living ones are rarely detected in either bottom seawater or deep-sea carbonatites [52].

### The co-option of conserved genes in guiding the development of bacteriocytes

As a specialized and stable niche for endosymbiosis, bacteriocytes are intriguing and crucial in understanding the evolution of symbiosis [2,6]. While previous studies demonstrated that the proliferation cells in the growth zones of gills can differentiate into bacteriocytes, our single-nucleus and spatial transcriptome data, for the first time as far as we know, traced the successive development trajectory of bacteriocytes. We provide evidence that proliferation cells and bacteriocytes can be divided into different states and that only a subset of proliferation cells are the progenitors of bacteriocytes. The differentiation and maturation of bacteriocytes may be guided by a set of mollusk-conserved transcription factors (including *ezh2*, *elf-3*, *znf271*, and *met*). Besides, the regulatory networks responsible for the development of bacteriocytes are likely to also control the immune and metabolic processes, two of the most noticeable features of bacteriocyte function. All these transcription factors are evolutionarily conserved across mollusks and are in agreement with the general host phylogeny. Additionally, although less investigated in mollusks, homologues of the majority of these genes, such as *ezh2,*  *elf-3*, *met*, and *mynn*, are all known as crucial regulators of cell proliferation and differentiation [53–56]. Among these genes, *ezh2* is of particular interest as it is ubiquitously expressed by all gill cell clusters (especially for bacteriocytes) and is drastically upregulated in state 3 of all proliferation cells. The expression pattern of *ezh2* suggests a leading role of histone methylation in controlling the function and development of bacteriocytes, which also supports the conclusion that the formation of symbiotic cells in deep-sea mussels is due to the co-option of conserved rather than the emergence of evolutionarily novel genes [57]. Similar phenomena have also been observed in the deep-sea scaly-foot snail *Chrysomallon squamiferum*, where the formation of a biomineralized skeleton is driven by an ancestral intrinsic toolkit conserved across mollusks [58].

### The possible role of symbionts in the function and development of bacteriocytes

Notably, we also found that the function and development process of bacteriocytes could respond to the symbiont losses induced by methane reduction. The global decline of bacteriocyte marker genes and increase of cells in an immature state in decolonized mussels demonstrate the phenotypic plasticity of symbiotic cells and suggest the participation of symbionts in regulating the function and development of molluscan bacteriocytes. Symbiont participation in the development of symbiotic cells and organs has attracted increasing attention in recent years but has remained largely unclear at the mechanistic level [59,60]. Here, we found that the *cyp39a1* gene and a crucial nuclear receptor gene (*nhr-23*) were differentially expressed during the maturation of bacteriocytes. In addition, up to seven genes involved in the sterol-related signaling pathway (including *cyp39a1* and six nuclear receptor genes) were also differentially expressed in the bacteriocytes of decolonized mussels, highlighting the possible participation of symbiont sterol metabolism in regulating the function and development of mussel bacteriocytes. In support of this hypothesis, we previously observed the suppression of sterol/steroid biosynthesis in symbiont-depleted deep-sea mussels after long-term atmospheric cultivation without methane supply [44]. Furthermore, other studies have also shown that symbiotic Wolbachia modulate host development and reproduction via the steroid-nuclear receptor signaling pathway [61]. Sterol metabolism in symbionts may therefore be a common mechanism in the development of symbiotic cells and organs since several animal hosts rely on their symbionts for sterol intermediates [62–64]. Besides sterol metabolism, it is noteworthy that glucose and ammonia metabolism could also serve as intracellular signals of symbiosis-related processes (*e.g.*, via mTOCR1-mediated symbiont digestion) [65–68]. Although the regulatory role of symbionts in the function and development of symbiotic cells remains to be fully elucidated, the symbiont-mediated phenotypic plasticity in molluscan bacteriocytes could benefit the host to respond rapidly and globally to the fluctuating environment of the deep sea, especially when the methane supply changes. In addition, the coordinated regulation of endocytosis and digestion, as well as the metabolic interactions with symbionts, may also help the mollusk host to reshape the symbiotic associations according to the local environment, which therefore further assists in their presence across a wide range of deepsea habitats [69–71]. The dynamic plasticity in the function and development of molluscan bacteriocytes, especially the global repression of marker genes and increase in the number of cells in an immature state in decolonized mussels, also supports the idea that maturation is not a single-ended process but a dynamic continuum of adaptive states set by genes and the environment [72].

There are also some limitations to the present study. For example, although spatial transcriptome analysis was conducted, the relationship between gene expression and cell location could not be addressed due to the limited resolution of ST-seq data (55 µm in diameter per spot with a 100 µm center-to-center distance between spots). The spot size in ST-seq may also aggregate signals from multiple cells, thereby compromising single-cell resolution, despite its integration with snRNA-seq data. In addition, some of the hypotheses raised need to be verified in future studies using mussel samples in various symbiotic states, including aposymbiotic, recolonized, post-larval, and juvenile mussels, and employing new experimental paradigms (such as high-resolution ST-seq and scRNA-seq). Nevertheless, by combining single-nucleus and spatial transcriptome data, a phagocytosis assay, an EdU assay, and 3D electron microscopy data, we successfully established a pipeline to reveal the molecular functions and development of bacteriocyte lineages in non-model deep-sea mussels. Our results indicate that the function and development of bacteriocytes are highly coordinated through the co-option of conserved genes and dynamically modulated by symbionts. In addition, it was surprising to find that both endosymbionts and bacteriocytes persisted even after the two-year transplantation to a low methane site, where the methane concentration is 39-fold lower than in the seepage region, a level similar to that in regions with massive piles of dead mussels. Meanwhile, the immune and metabolic processes within mussel bacteriocytes could be dynamically modulated to sustain long-lasting symbiotic associations, thereby enhancing the resilience of deep-sea mussels to methane reduction. Collectively, the intimate symbiotic associations and robust plasticity of symbiotic cells have likely made deep-sea mussels one of the most successful organisms in the deep sea.

## Materials and methods

### Experimental design

The goal of the present study was to address the molecular function and developmental trajectory of bacteriocytes in the deep-sea mussel *G. platifrons*. To achieve this goal, we employed methanotrophic *G. platifrons* collected from cold seeps as our model, and used spatial transcriptomics, single-nucleus transcriptomics, and meta-transcriptomics to characterize both the bacteriocytes and endosymbionts. We also employed an *in situ* transplantation assay to generate decolonized mussels and to elucidate the dynamic responses of symbiotic cells in response to symbiont depletion. We used EdU-labeling, phagocytosis assays, FISH assays, IF assays, TEM, 3D electron microscopy, and phylogenetic analysis to verify hypotheses obtained from sequencing data.

### *In situ* transplantation and animal collection

*G. platifrons* mussels were collected from the cold seeps (22°06′N, 119°17′E) of the South China Sea during the cruises of 2018 (cruise *KEXUE*-1807) and 2020 (cruise *KEXUE*-2002). Permits for the cruises and sample collections were granted by the Institute of Oceanology, Chinese Academy of Sciences. Mussel samples were collected on dives 181, 206, 231, and 241 by remotely operated vehicle (ROV) FAXIAN. The mussel fauna lives at 1120 m beneath the surface at a temperature of approximately 3.35°C, salinity of approximately 35.54 psu (practical salinity unit), dissolved oxygen of 2.98–3.17 mg/l, and methane concentrations of up to 31,227 ppm in the seepage region [73]. To protect the specimens from temperature and pressure fluctuations during sampling, all mussel samples were collected using our self-designed isothermal isobaric sampler and a self-designed manually controlled macrofauna *in situ* sampling device as described previously [43]. The self-designed isothermal isobaric sampler consists of two parts. The inner part is a titanium lumen that can protect the samples from depressurization (filled with seawater and therefore can keep the mussel alive during sampling). The outer part of our sampler is an acrylic cabin with replaceable ice bags that can prevent heat exchange with the surrounding seawater during sample recovery. After retrieving and depressurizing the isothermal isobaric sampler, the mussels were immediately dissected into two halves to remove excess seawater and quickly frozen in an isopentane bath (Catalog No. M813375, Macklin, Shanghai, China) with liquid nitrogen for snRNA-seq and ST-seq. The macrofauna *in situ* sampling device was installed on the ROV platform and manually controlled by the ROV pilot. Mussel samples can be placed into the *in situ* sampling device for immediate treatment with RNAsafer stabilizer reagent or other reagents to minimize expression alterations of samples during recovery. Briefly, a total of 20 mussels living in close proximity to active fluid seeps were collected as representatives of normal mussels (designated as the InS group). Among them, seven mussels were collected using an isothermal isobaric sampler in 2018, seven were collected using a multipurpose *in situ* sampling device in 2020 after being treated with RNAsafer stabilizer reagent (Catalog No. R0424, Omega Bio-Tek, Norcross, GA) *in situ*, and the remaining six were collected using a multipurpose *in situ* sampling device after being treated with 4% paraformaldehyde *in situ*. Three mussels from the InS group were used for snRNA-seq (Chromium platform of 10x Genomics, Pleasanton, CA). One of these three mussels was also subjected to spatial transcriptome sequencing (ST-seq on the Visium platform of 10x Genomics). All 14 mussels collected by the isothermal isobaric sampler or after RNA-stabilizing treatment were also subjected to meta-transcriptomic sequencing. Mussels collected after paraformaldehyde treatment were subjected to ISH, IF, and TEM imaging. During the 2018 cruise, we also conducted an *in situ* transplantation assay, translocating dozens of mussels from the seepage region to an authigenic carbonate region with a low concentration of CH_4_ (at approximately 100 m from the seepage, the methane concentration decreased to 800 ppm). Among these transplanted mussels, five were retrieved 604 days later during the 2020 cruise and designated as the DeC group. For these retrieved mussels, one was collected using an isothermal isobaric sampler and then employed for snRNA-seq, ST-seq, and meta-transcriptome. Four were collected with the aforementioned multipurpose *in situ* sampling device (treated with RNAsafer stabilizer reagent) and subjected to meta-transcriptome.

### snRNA-seq

Four individual mussels of similar size (approximately 80 mm in length) from the InS and DeC groups were used for snRNA-seq. Considering the difficulties involved in the isolation and *in vitro* culture of gill cells, snRNA-seq analysis of *G. platifrons* gill tissue was conducted using nuclei isolated from fresh frozen samples collected in an isothermal isobaric manner to minimize the potential effects on gene expression caused by mussel sampling and single cell preparation, and to maintain consistency with our previous study. The isolated nuclei were assayed following the 10x Genomics single-cell protocol by generating single-nucleus gel bead-in-emulsion (GEM) on a GemCode single-cell instrument (https://www.10xgenomics.com/support). Full-length cDNA synthesis was performed using Chromium Next GEM single cell 3′ reagent kit (v3.1) and then sequenced on an Illumina HiSeq X Ten platform (Gene Denovo Biotechnology, Guangzhou, China).

### Cryosectioning and ST-seq

To help determine the taxonomy of all gill cells, including bacteriocytes, we also conducted ST-seq with a cross-sectioned middle region of the gill (ventral view) from the InS group. The gill tissue for 10x Visium ST-seq was first incubated with precooled methanol (4°C) for 15 min to reduce potential damage from the cryostat blade during cryosectioning. The tissue was then embedded in O.C.T. compound (Catalog No. 4583, Sakura, Torrance, CA) and cross-sectioned at 10 µm through the middle part of the gill using a cryostat (Leica CM1950, Heidelberger, Germany). Gill sections were then stained with 4′,6-diamidino-2-phenylindole (DAPI, Catalog No. P36935, Thermo Fisher Scientific, Waltham, MA) and hematoxylin and eosin staining (HE, Catalog No. E607317, Sangon Biotech, Shanghai, China), and the morphology of the gill-tissue sections was assessed under light microscopy (Nikon ECLIPSE Ni, Tokyo, Japan). Successive sections obtained from the embedded tissue blocks were placed on Visium spatial tissue optimization slides within the capture area for the tissue optimization assay. For the sequencing assay, the gill sections were first mounted on Visium spatial gene expression slides and permeabilized according to previously described parameters (permeabilization time of 9 min) after HE staining and imaging using a light microscope. After reverse transcription and library preparation, all samples were sequenced on an Illumina HiSeq X Ten platform (Gene Denovo Biotechnology). The remaining sections of the same tissue were further subjected to ISH assays to aid cell type annotation.

### Cell clustering, cell type annotation, and analysis of differentially expressed genes

Cell clustering of *G. platifrons* gill tissue was first performed using snRNA-seq-derived data. After quality control, all raw reads were mapped onto a *G. platifrons* reference genome. The genome was first reported by Sun et al. [19] and updated recently with Hi-C and high-depth PacBio long-read sequencing by us (GenBank accession NO. JAOEFJ000000000). Unique molecular identifiers (UMIs) in each sequenced read were counted and corrected for sequencing errors. Using valid barcodes that were identified based on the EmptyDrops method [74], the gene matrices of all cells were produced and imported into Seurat (v3.1.1) for cell clustering using a graph-based clustering approach [75].

Single-cell clustering of *G. platifrons* gill tissue was also performed using ST-seq-derived data, with the advantage of cell type annotation. A slide image obtained before permeabilization was first imported into Space Ranger software (https://www.10xgenomics.com/support/software/space-ranger/) for fiducial and Visium barcoded spot alignment. After decoding correlations between tissue, capture spots, and barcodes, a splice-aware alignment of sequencing reads to the *G. platifrons* genome was performed using STAR in the Space Ranger software package.

An integrated analysis of data derived from snRNA-seq, ST-seq, and *in situ* hybridization assay was then used for cell type annotation in the InS group. Briefly, anchor-based integration was first used to integrate ST-seq data with snRNA-seq data using the FindIntegrationAnchors command in Seurat-(v3.2). All cell-type labels in snRNA-seq were then transferred to spatial data using the TransferData command. Cell type prediction scores indicating the similarity between ST-seq spots and snRNA-seq cell clusters were calculated simultaneously, and only spot-cluster pairs with the highest scores were considered for further cell-type annotation. For annotation and cell-type verification, cell types in gill tissue were inspected using HE-stained images of gill cryosections and tissue plots colored by the clustering of ST-seq data. Several cell markers obtained using the FindAllMarkers function in Seurat were also cloned and subjected to *in situ* hybridization using gill tissues from the InS group.

To further investigate the biological function of all identified cell clusters, differentially expressed genes (DEGs) were surveyed in both snRNA-seq and ST-seq data. For snRNA-seq, expression values of all identified genes in a given cluster were compared against those of the other cell clusters using the Wilcoxon rank sum test as suggested recently [76]. Genes expressed mainly in the target cluster (more than 25% of cells were designated as a target cluster), and showing at least 1.28-fold upregulated expression levels and *P* < 0.01, were considered DEGs per cell cluster. According to previous studies [77–79], model-based analysis of single-cell transcriptomics (MAST) in the R package was used to determine DEGs in a single cell cluster using the same criteria as those used for snRNA-seq. All genes in ST-seq data were further analyzed for spatially-specific DEGs using markvariogram in the Seurat R package and mark-segregation hypothesis testing in the trendsceek R package. Genes with “r.metric.5” parameter values of < 0.8 and *P* < 0.01 were designated as spatially DEGs.

### Meta-transcriptome sequencing

To explore the expression atlas of symbionts in the InS and DeC groups, we performed meta-transcriptome sequencing using 14 samples from the InS group and five samples from the DeC group as described previously. Total RNA extraction, removal of eukaryotic and prokaryotic rRNA, and synthesis of cDNA libraries using bulk-seq were conducted as described previously [43]. cDNA libraries from the InS and DeC groups were finally sequenced on an Illumina HiSeq 2500 platform with paired-end reads performed by Novogene (Tianjin, China). After quality control, the filtered reads were aligned against both the endosymbiont and *G. platifrons* genomes using HISAT (v2.0.4) [80]. The genome of methanotrophic symbionts was first reported by Takishita et al. [33], and we updated it with high-depth PacBio long-read sequencing [raw data deposited in National Center for Biotechnology Information (NCBI) with accession No. PRJNA 893022] [81]. After mapping the raw reads to the combined pan-genome of the host and symbiont, the reads originating from either the host or the symbiont were extracted and treated separately for expression analysis. The expression levels of host and symbiont genes were calculated using HTSeq (v0.6.1) [82], while significant differences between groups were determined using DESeq2 (v1.10.1) [83]. Additionally, the top 10% of genes with the most abundant mRNA transcripts in the InS group were designated as abundantly expressed genes to survey the biological processes that actively occurred under normal conditions *in situ*. For the PCoA analysis, the Bray-Curtis distance between samples was first calculated and subjected to subsequent analysis and visualization.

### GO/KEGG analysis, cell trajectory construction, and WGCNA of bacteriocytes

GO annotation of *G. platifrons* genes was obtained using Blast2GO software (v5.2) [84] and employed for GO enrichment analysis using homemade scripts. The number of DEGs for every GO term was first calculated, and the significantly enriched GO terms were determined using a hypergeometric test (FDR < 0.05). Kyoto Encyclopedia of Genes and Genomes (KEGG) pathway enrichment analysis was conducted using in-house scripts with KEGG annotations obtained from the KEGG database (http://www.genome.jp/kegg/pathway.html).

Single cell trajectory analysis was conducted using highly variable features (genes) identified by Seurat and Monocle (v2.6.4; https://github.com/cole-trapnell-lab/monocle-release) in a reversed graph embedding algorithm, and confirmed by Slingshot (https://github.com/kstreet13/slingshot), Cytotrace (https://cytotrace.stanford.edu/), PAGA analysis by Scanpy (v1.6.0; https://scanpy.readthedocs.io/en/stable/), and RNA velocity analysis by scVelo (https://scvelo.readthedocs.io/en/stable/). The gene expression matrix of proliferation cells and bacteriocytes was used in the analysis and visualized using the orderCells function of Monocle (sigma = 0.001, lambda = NULL, param. gamma = 10, tol = 0.001). For the PAGA analysis, the connectivity of each cell cluster was calculated based on the partition-based graph abstraction algorithm. After cell embedding with ForceAtlas2, the pseudo-time value of each cell was then calculated with the DPT algorithm. For the RNA velocity analysis, loom files were generated using velocyto (v0.17.3) and were subsequently merged using the combine function from the loompy Python module. An AnnData object was then created using the scVelo Python module. After identifying the highly variable genes, the velocities (directionalities) were computed based on the stochastic model and projected onto the UMAP embeddings generated from Seurat. Slingshot and Cytotrace analyses were conducted in R with default parameters and using snRNA-seq data generated from Seurat. WGCNA was conducted using the WGCNA package (v1.47; https://cran.r-project.org/web/packages/WGCNA/index.html) in R with gene expression values of power = 7 and minModuleSize = 50, and visualized using Cytoscape (v3.8.2; https://cytoscape.org/).

### EdU-labeling, phagocytosis, ISH assays, IF assays, 3D electron microscopy, quantitative real-time PCR, and phylogenetic analysis

The EdU-labeling assay was carried out *in situ* using mussels collected from the seepage region. Briefly, mussels were incubated with 5-ethynyl 2′-deoxyuridine (EdU, final concentration of 40 µM; Catalog No. E10415, Thermo Fisher Scientific) in a self-designed, manually controlled macrofauna *in situ* experiment device for approximately 18 h, and then retrieved using an isothermal isobaric sampler. The click-iT plus EdU imaging kit (Catalog No. C10337, Thermo Fisher Scientific) was then used to visualize the EdU signal.

Phagocytosis assay was conducted using primary gill cells obtained from the freshly collected mussels using previously described methods with modifications [39,85]. Because endosymbiotic methanotrophs are nonculturable, *Vibrio alginolyticus*, an environmental bacterium isolated from cold seeps, was used in this assay and labeled with fluorescein isothiocyanate (FITC; Catalog No. 104157, Sigma, St. Louis, MO). To collect primary gill cells, gill tissue was first treated with 1% trypsin (diluted in sterilized seawater) at 4°C for 30 min, and then centrifuged at 300 *g* (4°C, 5 min) and 800 *g* (4°C, 5 min). Cell pellets were resuspended in modified L15 medium (Catalog No. 11415064, Gibco, Carlsbad, CA; supplemented with 0.54 g/l KCl, 0.6 g/l CaCl_2_, 1 g/l MgSO_4_, 3.9 g/l MgCl_2_, and 20.2 g/l NaCl) to a final concentration of 1 × 10^6^ cells/ml and incubated with the same volume of FITC-labeled *V. splendidus* (1 × 10^8^ cells/ml) for 30 min at 4°C in the dark. Primary cells were then washed three times with modified L15 medium to remove extracellular bacteria. Cells were stained with DAPI and DiI perchlorate, and imaged using a laser scanning confocal microscope (Zeiss LSM710, Jena, Germany).

FISH and double-label FISH assays were conducted as described previously [24,28]. For cell-type verification, cell markers were cloned using gene-specific primers to synthesize FISH probes. FISH analysis of symbionts was performed using a cyanine 3 (Cy3)-labeled Eub338 eubacteria probe (5′-GCTGCCTCCCGTAGGAGT-3′) or an FITC-labeled pmoB (methanotroph-specific gene) probe (5′-CGAGATATTATCCTCGCCTG-3′) or a digoxigenin (DIG)-labeled pmoB probe. For the double-label FISH assay of bacteriocytes, Gill sections were first hybridized overnight with fluorescein-labeled cell marker probes and DIG-labeled methanotroph-specific rRNA probe. After incubations with anti-fluorescein-peroxidase (POD) (Catalog No. 11426346910, Roche, Basel, Switzerland) or anti-DIG-POD (Catalog No. 11207733910, Roche), the fluorescent signals of cell marker probes and methanotroph probes were developed by Alexa Fluor 488 tyramide reagent (Catalog No. B40932, Thermo Fisher Scientific) and Alexa Fluor 594 tyramide reagent (Catalog No. B40935, Thermo Fisher Scientific) according to the manufacturer’s protocol, respectively. After mounting with DAPI, gill tissues from both FISH and double-label FISH assays were visualized and imaged using a laser scanning confocal microscope (Zeiss LSM710).

For the IF assay, unique peptide fragments of 24-hydroxycholesterol 7 alpha-hydroxylase CYP39A1, sugar phosphate exchanger SLC37A2, and ammonium transporter *RHBG-A* were first synthesized by Sangon Biotech (Shanghai, China) and employed as antigens to produce rabbit polyclonal antibodies. After antigen affinity purification and specificity verification by enzyme-linked immunosorbent assay (ELISA) and Western blotting, the antibodies were then used in an IF assay using paraffin-embedded gill tissue (5 μm) according to methods described previously [24], with the assistance of ServiceBio (Wuhan, China). Specifically, an anti-lipid A antibody (Catalog No. ab8467, Abcam, Cambridge, MA) was used to indicate endosymbionts. After incubation with FITC- and Cy3-labeled secondary antibodies (Servicebio, Wuhan, China), gill sections were visualized and imaged using a laser scanning confocal microscope (Zeiss LSM710).

For the 3D electron microscopy assay, gill tissues collected from adult mussels (obtained isothermally) were fixed with paraformaldehyde-glutaraldehyde, treated with 1.0% osmium tetroxide (OsO_4_), and infiltrated with acrylic resin. The embedded tissues were then serially sectioned at 100 nm using an ultramicrotome (EM UC7, Leica, Vienna, Austria) and mounted onto a silicon wafer before imaging. The scanning electron microscope micrographs of the mounted sections were captured using Helios NanoLab 600i FIB-SEM (FEI, Hillsboro, OR). The obtained serial section images were aligned in Amira (v2019.3; https://www.thermofisher.com/hk/en/home/electron-microscopy/products/software-em-3d-vis/amira-software.html) for 3D reconstruction of bacteriocytes and cell proliferation analysis.

Estimation of the symbiont abundance in both the InS and DeC groups was conducted using a quantitative real-time PCR assay according to the method described previously [86]. Briefly, genomic DNA from the gill tissue was extracted using the E.Z.N.A. Mollusc DNA kit (Catalog No. D3373, Omega Bio-Tek). Symbiont abundance was then calculated by comparing the relative DNA content of the symbiont methane monooxygenase gene with that of the internal control host genes (β*-actin*) using quantitative real-time PCR. The primers used in the assay were as described in our previous study [86]. All data were normalized using the 2^−ΔΔCt^ method and subjected to one-way analysis of variance (ANOVA) in SPSS (v20; https://www.ibm.com/spss) to detect significant differences between samples.

For phylogenetic analysis of hub transcription factors, homologous proteins were first obtained using the National Center for Biotechnology Information NCBI BLASTp and aligned using SeaView [87]. Maximum likelihood phylogenetic trees of these proteins were then constructed using MEGA software (v11) in the Jones-Taylor-Thornton model with a bootstrap of 100.

## Supplementary Material

qzaf109_Supplementary_Data

## Data Availability

All sequencing data have been deposited in the NCBI (BioProject: PRJNA838712), which are publicly accessible at https://www.ncbi.nlm.nih.gov/bioproject/. The data have also been deposited in the BioProject database at the National Genomics Data Center (NGDC), China National Center for Bioinformation (CNCB) (BioProject: PRJCA036932), which are publicly accessible at https://ngdc.cncb.ac.cn/bioproject/. Raw images for 3D electron microscopy have been deposited at Figshare (https://figshare.com/articles/figure/Raw_image_for_3D_electron_microscopy_of_mussel_gill/23575734).

## CRediT author statement

**Hao Chen:** Conceptualization, Writing – original draft, Funding acquisition, Project administration. **Mengna Li:** Methodology, Investigation, Writing – original draft. **Zhaoshan Zhong:** Methodology, Investigation, Resources. **Inge Seim:** Writing – review & editing. **Minxiao Wang:** Resources, Funding acquisition, Writing – review & editing. **Chao Lian:** Resources, Methodology, Investigation. **Lianhong Zhuo:** Investigation, Validation, Visualization. **Xinjiang Wan:** Methodology, Validation. **Hao Wang:** Methodology, Validation. **Guanghui Han:** Methodology, Validation. **Li Zhou:** Methodology, Investigation. **Huan Zhang:** Methodology, Software. **Lei Cao:** Methodology, Investigation. **Chaolun Li:** Supervision, Funding acquisition, Writing – review & editing. All authors have read and approved the final manuscript.
