# Supplementary material for: Function and Development of Deep-sea Mussel Bacteriocytes Revealed by snRNA-seq and Spatial Transcriptomics
Source: Genomics Proteomics Bioinformatics. 2025 Nov 25;24(1):qzaf109. doi: 10.1093/gpbjnl/qzaf109 (PMC13401447; doi:10.1093/gpbjnl/qzaf109)
Supplement: qzaf109_Supplementary_Data [file qzaf109_supplementary_data.zip › Supplementary material captions.docx]

**Supplementary material**

**Figure S1 PCoA and saturation analysis of transcriptome data**

**A.** Distribution pattern of endosymbionts (with Cy3-labeled Eub338 eubacteria probe, in red) in gill tissue of InS group. **B.** Distribution pattern of endosymbionts in gill tissue of DeC group. **C.** PCoA of the meta-transcriptome of the InS (isobaric samples, *n* = 7) and DeC groups (isobaric samples, *n* = 1, and RNAsafer-preserved samples, *n* = 4) using all mussel mRNA transcripts. **D.** PCoA of the meta-transcriptome of the InS (RNAsafer-preserved samples, *n* = 7) and DeC groups using all mussel mRNA transcripts. **E.** Saturation analysis of ST-seq data in the InS group. **F.** Saturation analysis of ST-seq data in the DeC group. **G.** Saturation analysis of snRNA-seq data in the InS group. **H.** Saturation analysis of snRNA-seq data in the DeC group. PCoA, principal co-ordinates analysis.

**Figure S2 Function enrichment analysis of gill cells, and phagocytosis and symbiont proliferation inside bacteriocytes**

**A.** The individual *t*-SNE projection of single-nucleus transcriptome of the InS (A1), DeC group (A2), and ST-seq of DeC group (A3) with color assigned by cell type. The integrated *t*-SNE projection of ST-seq of InS and DeC group (A4) with color assigned by cell type. **B.** Gene Ontology (GO) enrichment analysis of marker genes in bacteriocytes, DEPCs, PEBZCs and ICCs. Size of the dot represents number of enriched genes in pathway, and color represents q-value of enrichment result. **C.** Double FISH of bacteriocyte marker genes (fluorescein-labeled gene specific probe, green) and symbionts (DIG-labeled Eub338 eubacteria probe, orange) were conducted with DeC samples to verify the bacteriocyte cluster. **D.** Phagocytosis of FITC-labeled beads (2 μm in diameter) by bacteriocytes *in vitro* (Engulfed beads were labeled with triangle). **E.** Lysosome-mediated digestion (labeled with red triangle) and proliferation (labeled with black triangle) of endosymbionts inside bacteriocytes as revealed by 3D electron microscopy. More details for the lysosome-mediated digestion could be observed in Video S1. Details for the symbiont proliferation could be observed in Video S2.

**Figure S3** **Expression patterns of key bacteriocyte marker genes across distinct cell clusters of InS group**

The expression patterns of *CYP39A1* (**A**), *SLC37A2* (**B**), and *RHBG-A* (**C**) across distinct cell clusters, including bacteriocytes (circled with a black dashed line) were shown on the color-coded *t*-SNE projection of single-nucleus transcriptome of the InS group.

**Figure S4 Sterol biosynthesis pathways of endosymbionts and deep-sea mussel host**

**A.** Metabolic pathway of steroid biosynthesis in the symbiont as revealed using the genome (left) and meta-transcriptome (right, data from isobaric samples of the InS group, *n* = 7). For genomic data, all the identified genes are colored in grey. For meta-transcriptome data, all identified genes are colored in green, while abundantly expressed genes (top 10%) are colored in pink. **B.** Metabolic pathway of steroid biosynthesis in the host as revealed using the genome (left) and meta-transcriptome (right). For transcriptome data, only abundantly expressed genes (top 10%) are colored in green.

**Figure S5 Glucose/glycogen metabolic pathways of endosymbionts**

Glucose/glycogen metabolic pathways of the symbiont are colored based on the genome and meta-transcriptome data (isobaric samples of the InS group, *n* = 7). For genomic data, all the identified genes are colored in grey. For meta-transcriptome data, all identified genes are colored in green, while abundantly expressed genes (top 10%) are colored in pink.

**Figure S6 Glucose/glycogen metabolic pathways of mussel host**

Glucose/glycogen metabolic pathways of the host are colored based on the genome and meta-transcriptome data (isobaric samples of the InS group, *n* = 7). For genomic data, all the identified genes are colored in grey. For transcriptome data, only abundantly expressed genes (top 10%) are colored in green.

**Figure S7 Ammonia-related metabolic pathways of endosymbionts**

Ammonia-related metabolic pathways of the symbiont are colored based on the genome and meta-transcriptome data (isobaric samples of the InS group, *n* = 7). For genomic data, all the identified genes are colored in grey. For meta-transcriptome data, all identified genes are colored in green, while abundantly expressed genes (top 10%) are colored in pink.

**Figure S8 Differentially expressed genes analysis of bacteriocytes**

**A.** Differentially expressed genes of bacteriocytes in the InS group in comparison with the DeC group. **B.** GO enrichment analysis shows multiple metabolic pathways were modulated. Dot size represents the overall number of differentially expressed genes, up-down normalization = [number of upregulated genes] / [number of downregulated genes] − 1. **C.** Genes involved in carbohydrate, vitamin, lipid, and amino acid biosynthesis are mostly downregulated in decolonized mussels. **D.** Magnified images of gill tissue for the EdU labeling assay. Proliferating cells are labeled with EdU.

**Figure S9 WGCNA modules of gill tissue and the developmental process of bacteriocytes**

**A.** Clustering of WGCNA module eigengenes based on snRNA-seq data of the InS and DeC group. Eigengenes represent the overall expression levels of genes in each module of a given cell type and sample, with green indicating a negative correlation, and red indicating a positive correlation. **B.** GO enrichment analysis using element marker genes of bacteriocytes annotated from module Mod05. **C.** Development trajectories of bacteriocyte lineages revealed by PAGA (C1-2), Slingshot (C3) and Cytotrace (C4). For PAGA analysis, pseudo-time of different cell clusters is indicated with different color (C1). A Successive development trajectory can be observed for all cell clusters (C2). Two development lineages were predicted in Slingshot analysis, where DEPCs and PEBZCs were predicted to be progenitor cell of bacteriocytes (BC). In Cytotrace analysis, a co-location between immature bacteriocytes (less differentiated) and proliferation cells was observed. **D.** A positive velocity from PEBZCs and DEPCs to bacteriocytes was also observed in RNA velocity analysis, providing evidence for the successive differentiation from proliferation cells, mainly DEPCs and PEBZCs, to bacteriocytes. **E.** Venn diagrams of marker genes in bacteriocytes (E1), DEPCs (E2), PEBZCs (E3), and VEPC (E4) along with highly expressed genes of corresponding cell types in states 1, 2, and 3. **F.** Expression patterns of key transcription factors in different states of bacteriocytes, DEPCs, VEPCs, and PEBZCs.

**Figure S10 Phylogenetic analysis of hub transcription factors**

**A.** Phylogenetic analysis of EZH2, ELF-3, ZNF271 and AUST2 proteins. **B.** Phylogenetic analysis of all hosts using cytochrome C oxidase subunit I (mitochondrial) proteins. ELF-3, ETS-related transcription factor; ZNF271, zinc finger protein 271; AUST2, autism susceptibility gene 2.

**Table S1 Statistical analysis of ST-seq and snRNA-seq data**

**Table S2 snRNA-seq cell markers**

**Table S3 Differentially expressed markers in the DeC and InS groups as assessed using snRNA-seq**

**Table S4 Feature count information of meta-transcriptome of host and symbionts**

**Table S5 WGCNA sample expression pattern**

**Table S6 WGCNA networks in bacteriocyte lineages**

**Table S7 GO enrichment of WGCNA and all other markers in bacteriocytes**

**Table S8 State-related differentially expressed genes in proliferation cells and bacteriocyte lineages**

**Video S1 3D electron microscopy on the lysosome-mediated digestion of symbionts inside bacteriocytes of InS group (lysosome labeled in yellow)**

The embedded tissues were serially sectioned to a thickness of 100 nm using an ultramicrotome and mounted onto a silicon wafer before imaging. The scanning electron microscope micrographs of mounted sections were captured using Helios NanoLab 600i FIB-SEM (FEI, Hillsboro, USA). The obtained serial section images were aligned in Amira (version 2019.3) for the 3D reconstruction of bacteriocytes.

**Video S2 3D electron microscopy on the proliferation of symbionts inside bacteriocytes of InS group (proliferating symbiont labeled in yellow)**

The embedded tissues were serially sectioned to a thickness of 100 nm using an ultramicrotome and mounted onto a silicon wafer before imaging. The scanning electron microscope micrographs of mounted sections were captured using Helios NanoLab 600i FIB-SEM (FEI, Hillsboro, USA). The obtained serial section images were aligned in Amira (version 2019.3) for the 3D reconstruction of bacteriocytes.

**Video S3 3D electron microscopy on the proliferation of bacteriocyte of InS group (dividing nucleus labeled in brown)**

The embedded tissues were serially sectioned to a thickness of 100 nm using an ultramicrotome and mounted onto a silicon wafer before imaging. The scanning electron microscope micrographs of mounted sections were captured using Helios NanoLab 600i FIB-SEM (FEI, Hillsboro, USA). The obtained serial section images were aligned in Amira (version 2019.3) for the 3D reconstruction of bacteriocytes.
