# Supplementary figures and images for: Function and Development of Deep-sea Mussel Bacteriocytes Revealed by snRNA-seq and Spatial Transcriptomics
Source: Genomics Proteomics Bioinformatics. 2025 Nov 25;24(1):qzaf109. doi: 10.1093/gpbjnl/qzaf109 (PMC13401447; doi:10.1093/gpbjnl/qzaf109)

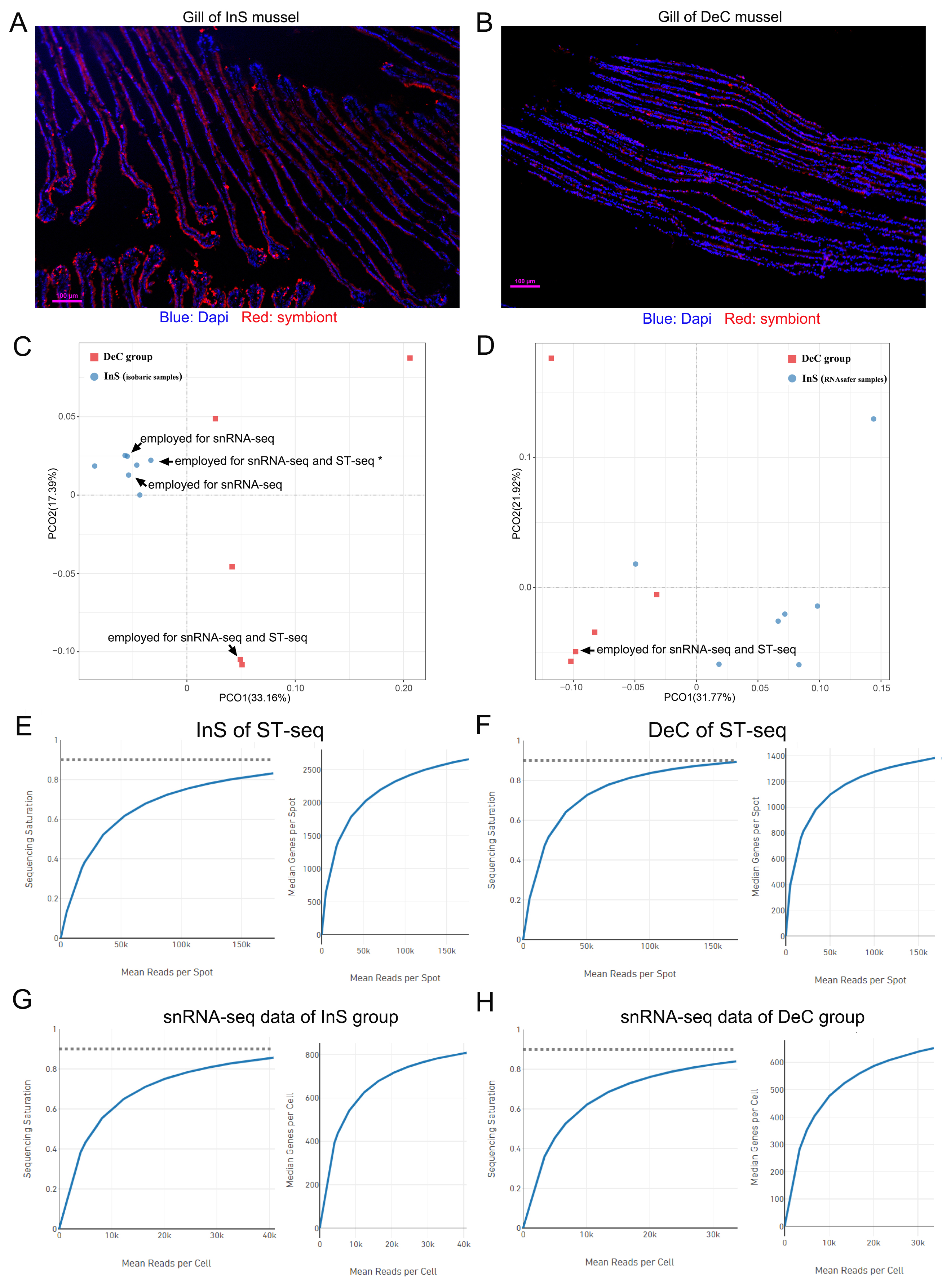

Supplement: qzaf109_Supplementary_Data [file qzaf109_supplementary_data.zip › Figure S1.jpg]

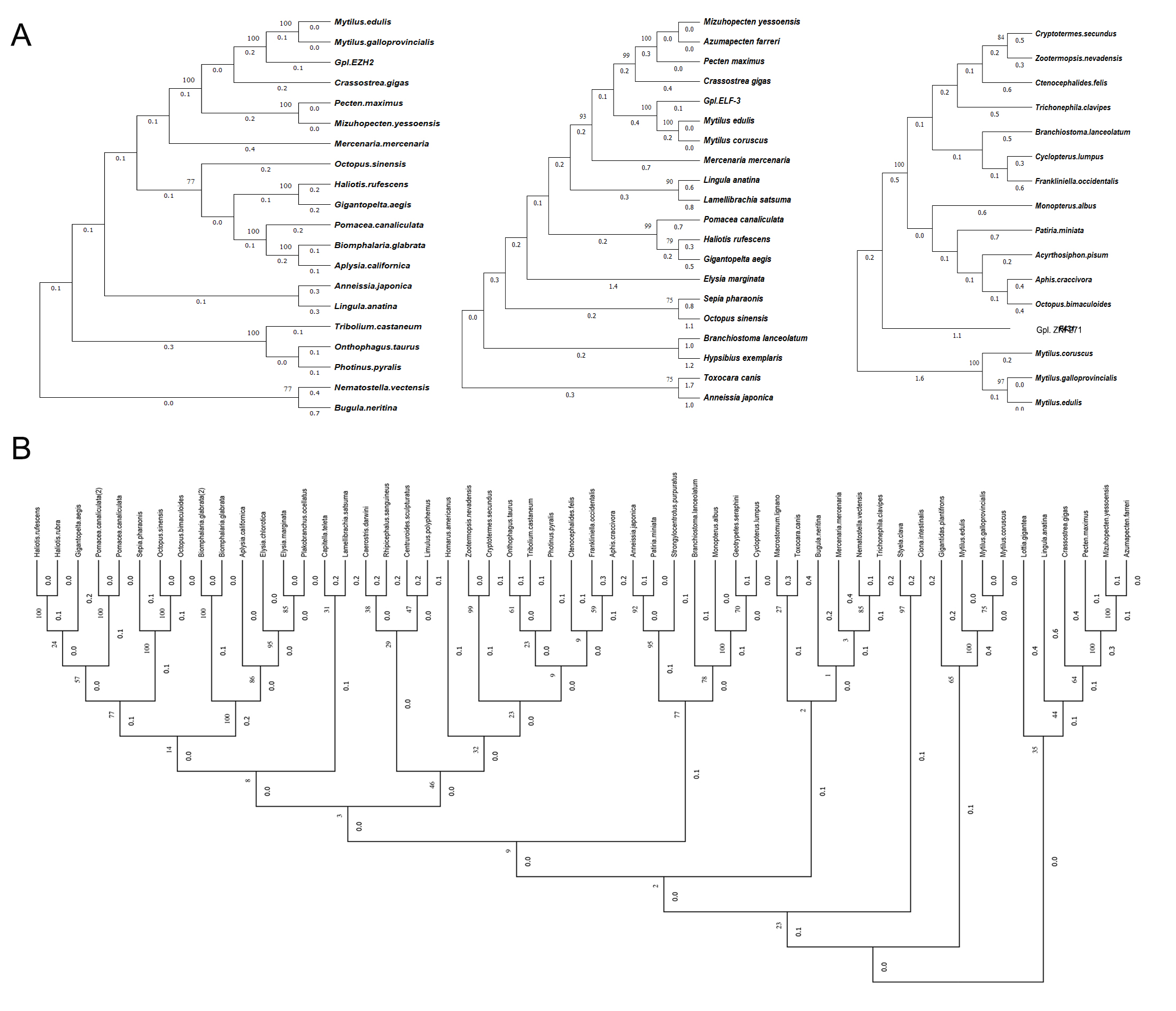

Supplement: qzaf109_Supplementary_Data [file qzaf109_supplementary_data.zip › Figure S10.jpg]

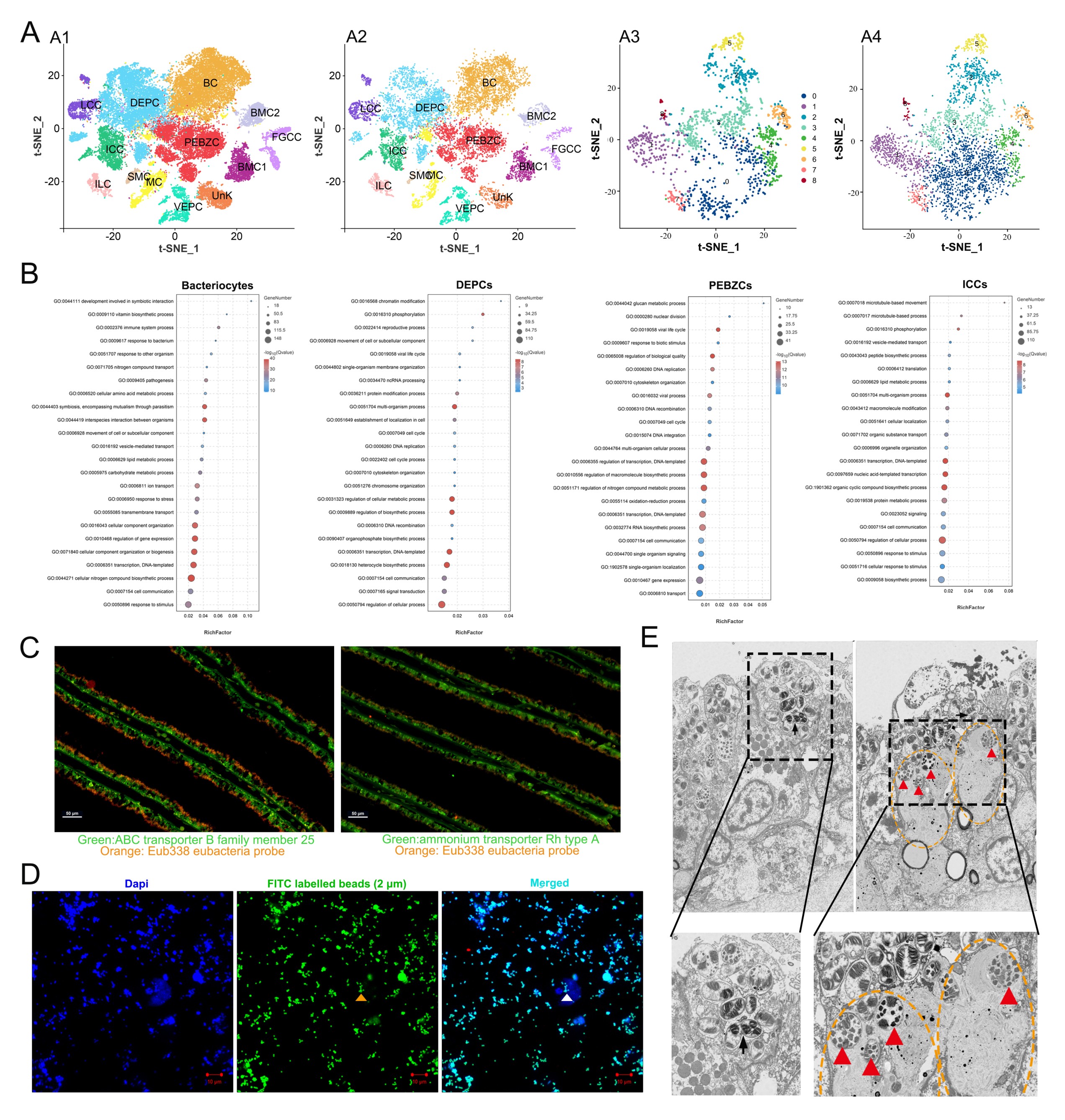

Supplement: qzaf109_Supplementary_Data [file qzaf109_supplementary_data.zip › Figure S2.jpg]

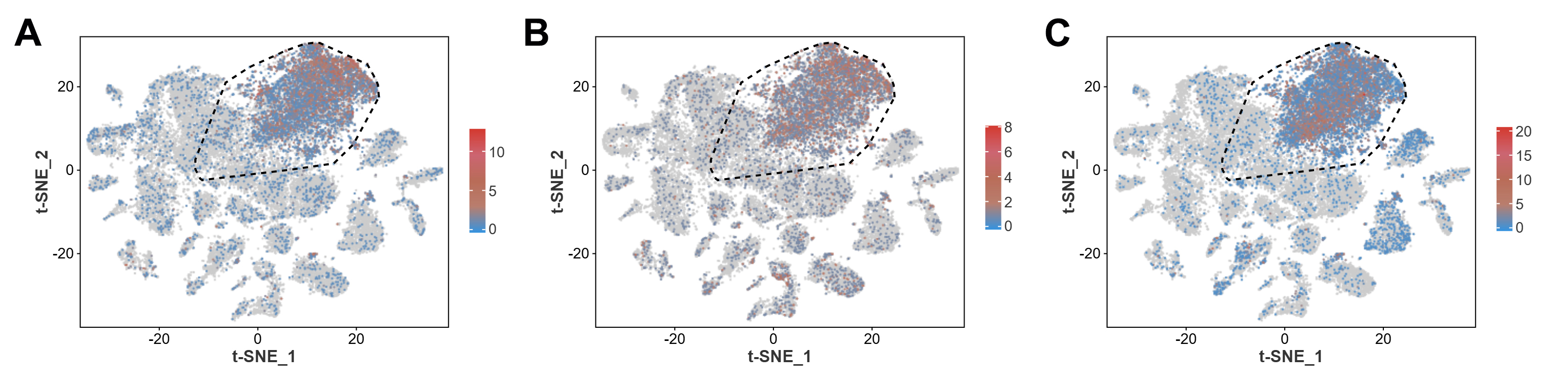

Supplement: qzaf109_Supplementary_Data [file qzaf109_supplementary_data.zip › Figure S3.jpg]

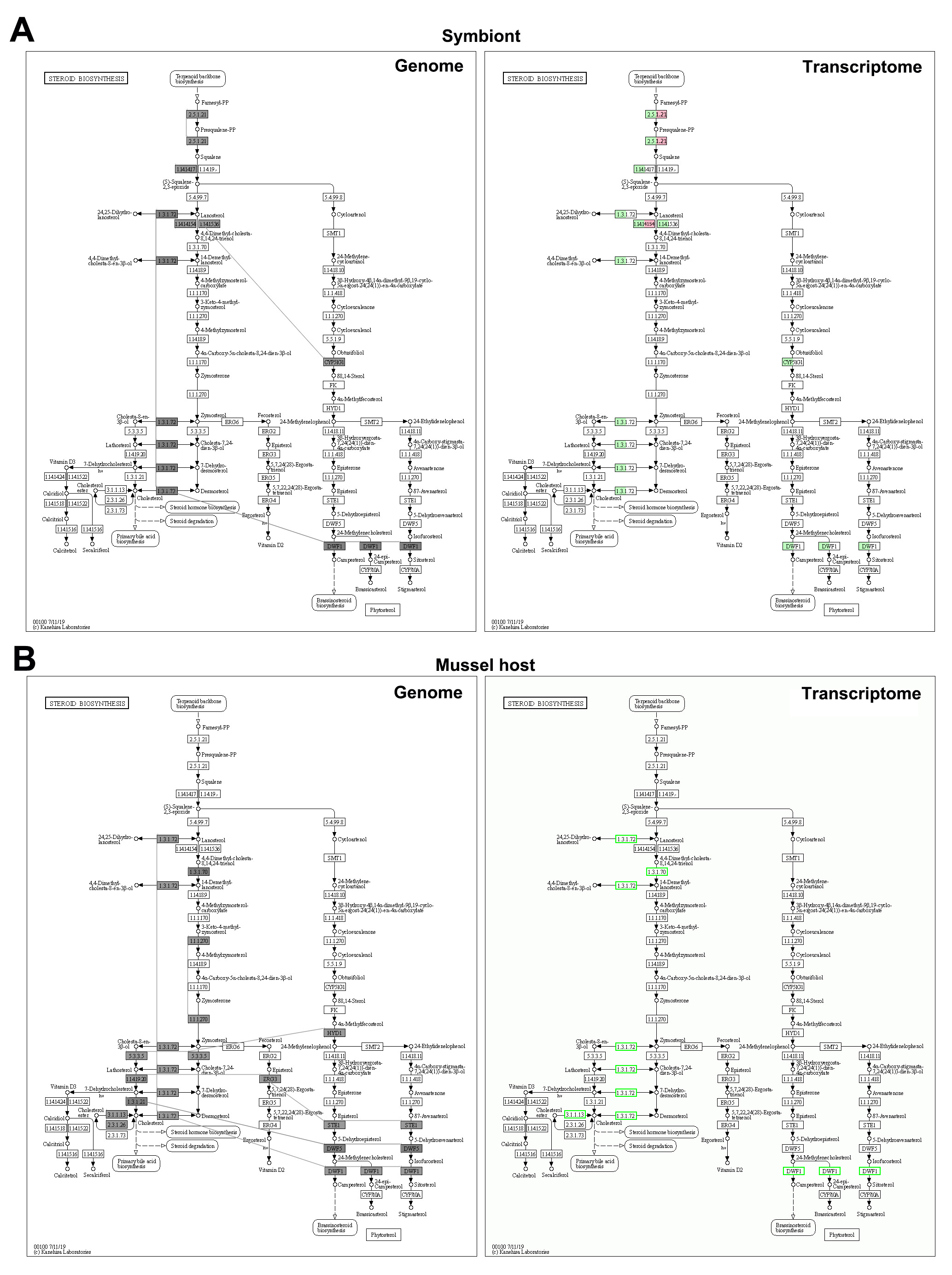

Supplement: qzaf109_Supplementary_Data [file qzaf109_supplementary_data.zip › Figure S4.jpg]

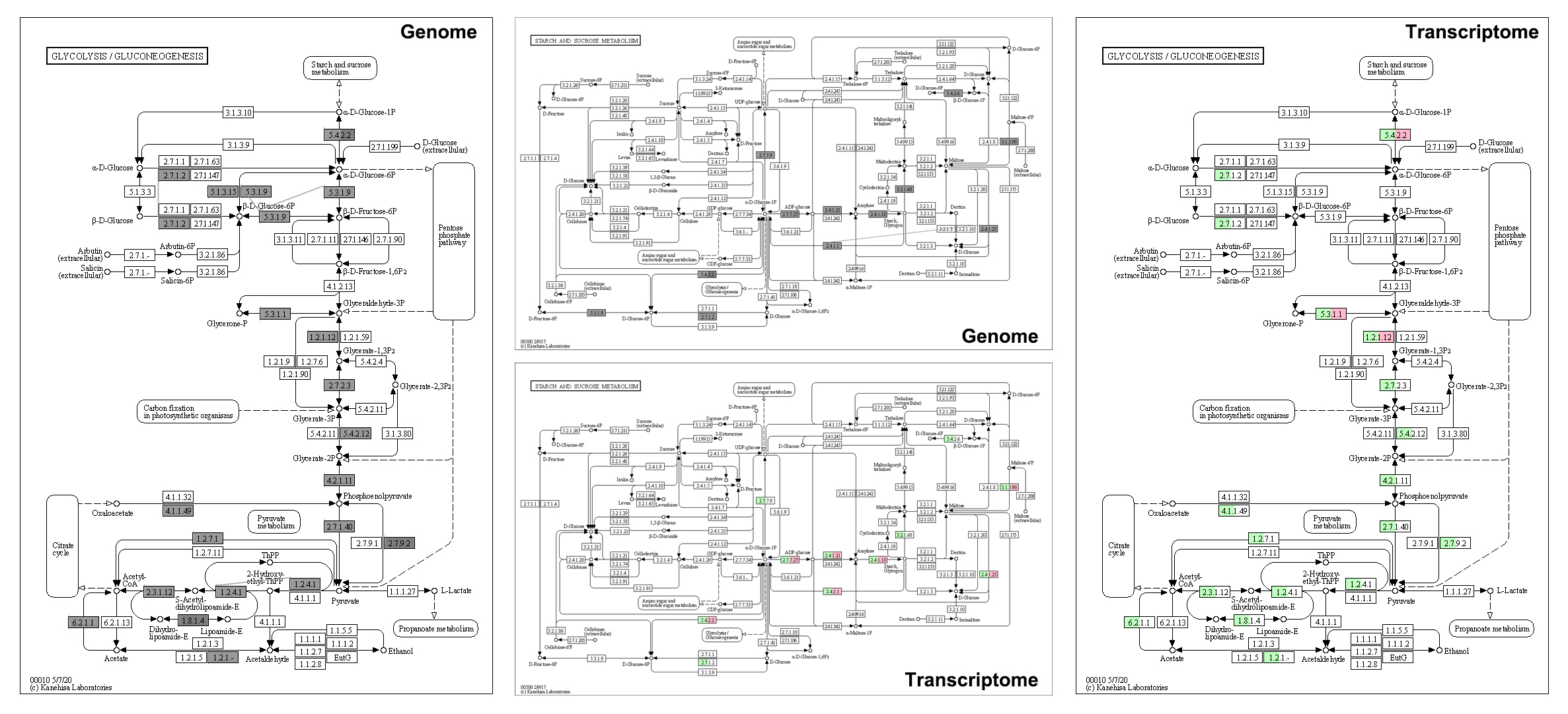

Supplement: qzaf109_Supplementary_Data [file qzaf109_supplementary_data.zip › Figure S5.jpg]

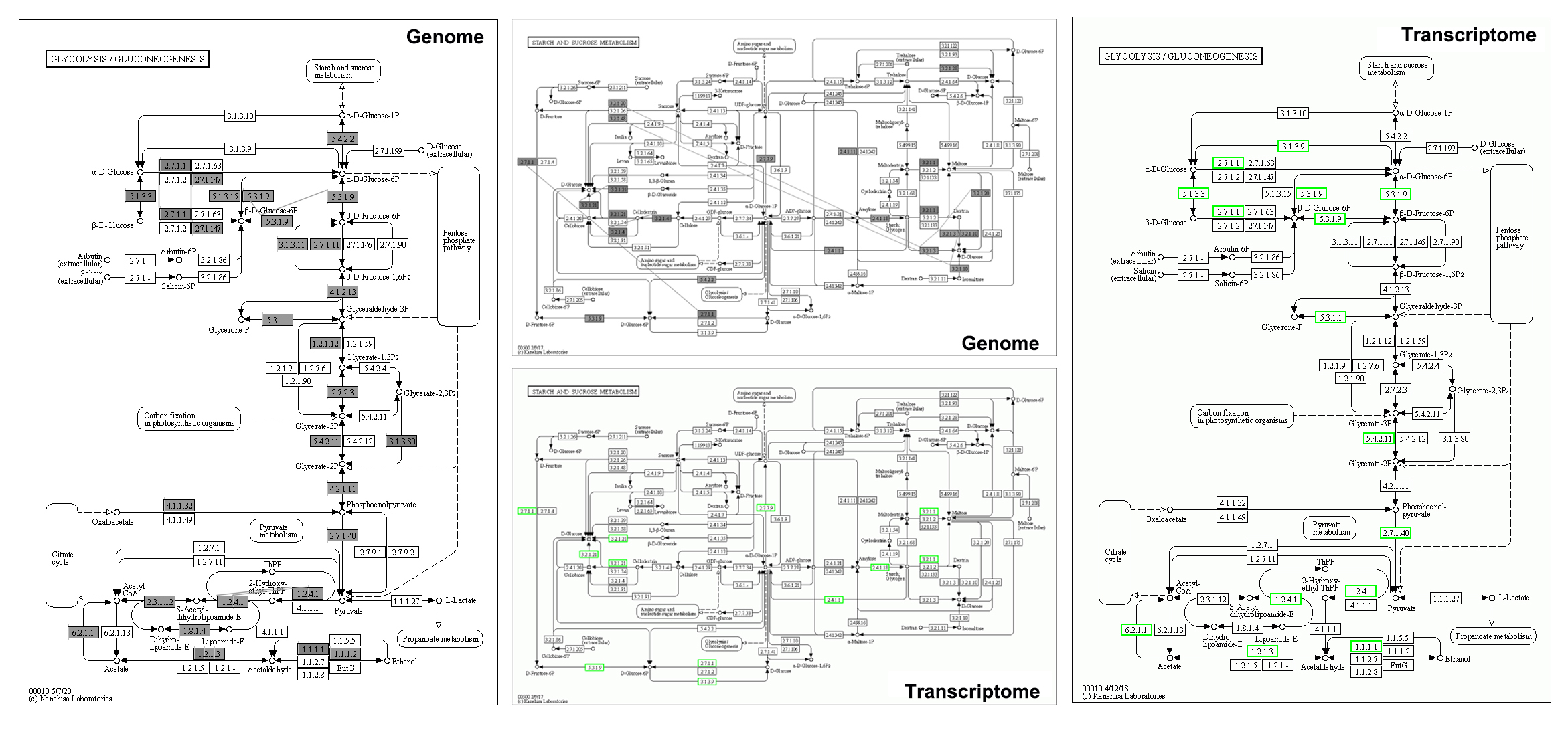

Supplement: qzaf109_Supplementary_Data [file qzaf109_supplementary_data.zip › Figure S6.jpg]

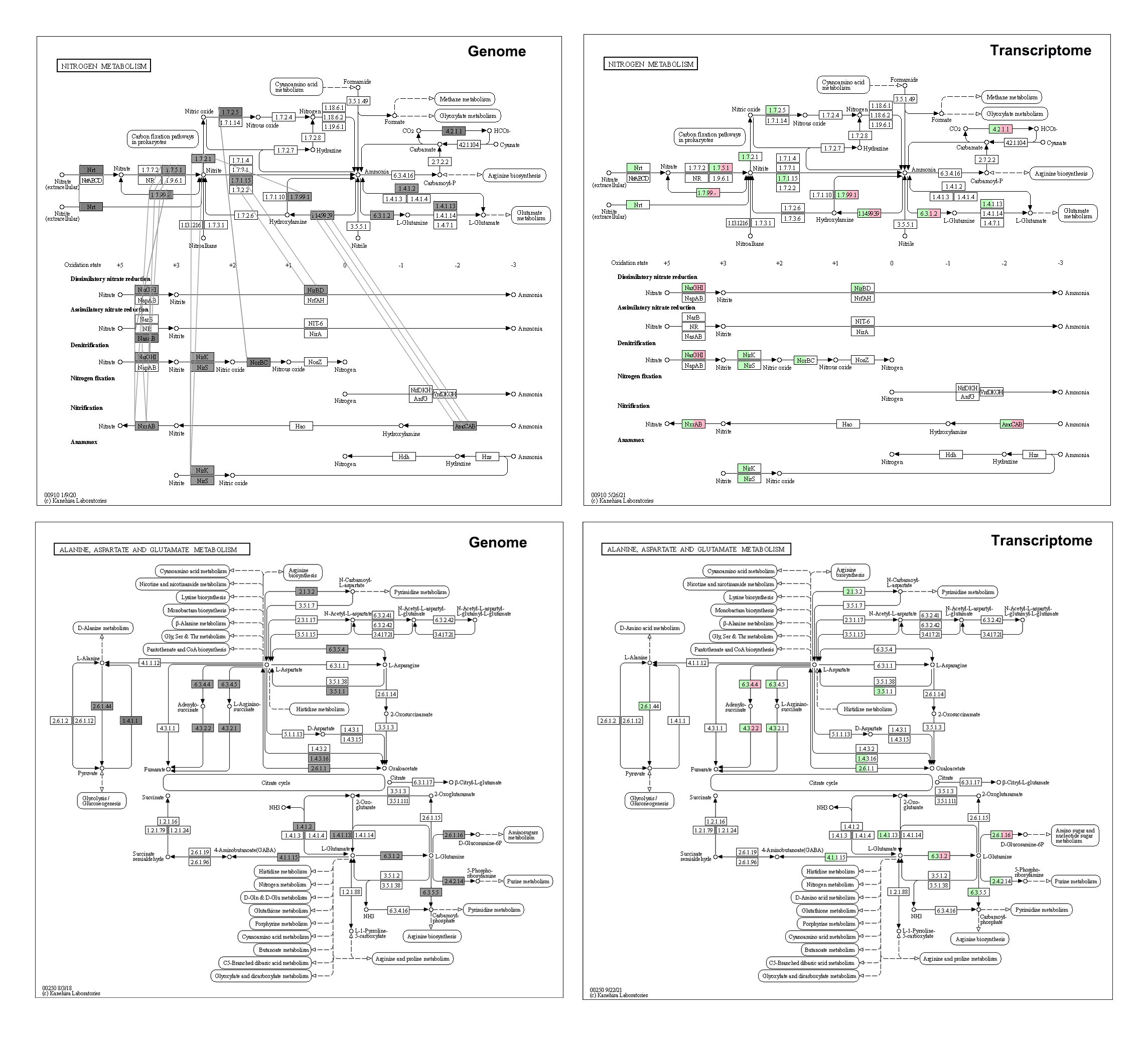

Supplement: qzaf109_Supplementary_Data [file qzaf109_supplementary_data.zip › Figure S7.jpg]

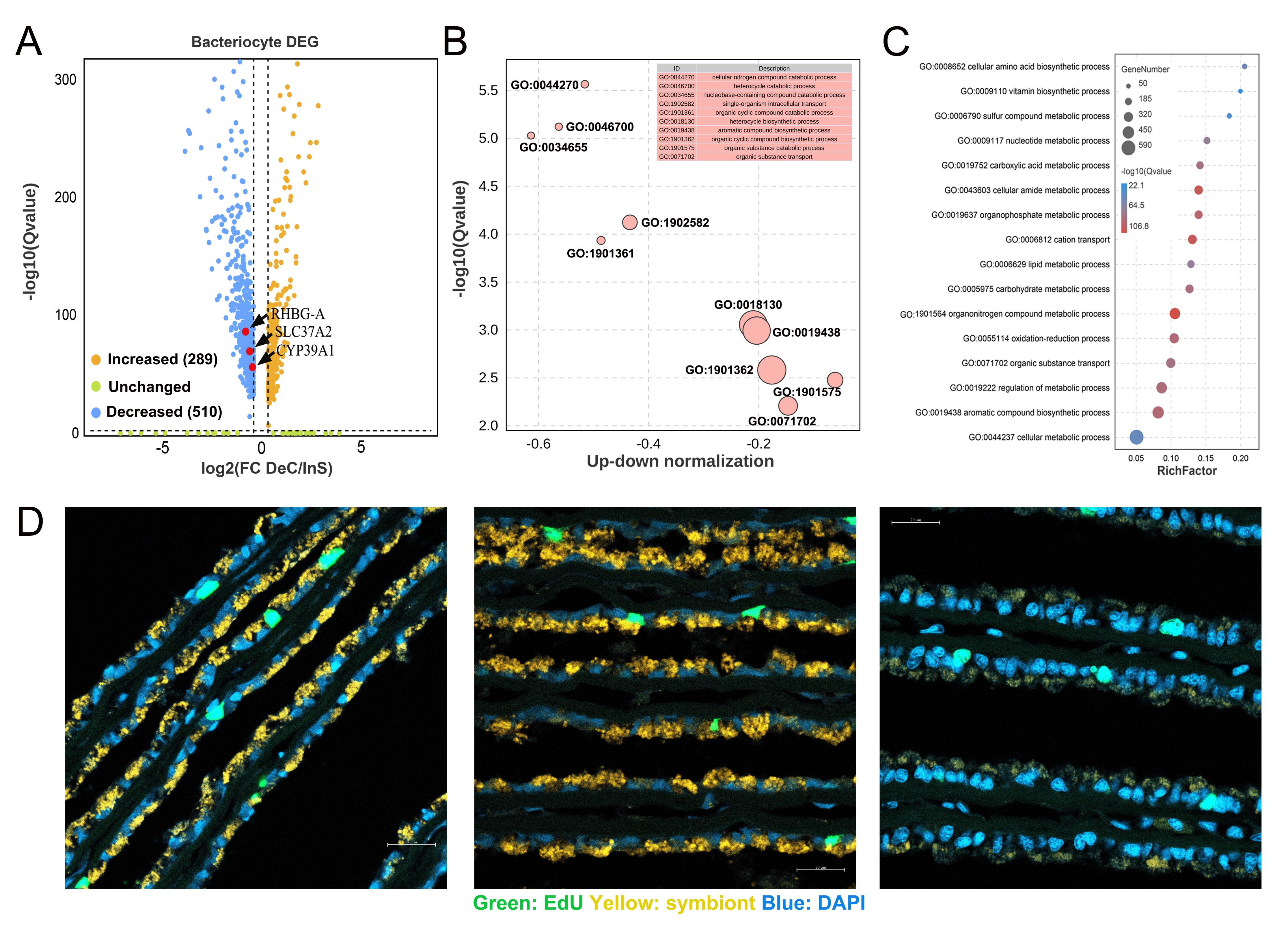

Supplement: qzaf109_Supplementary_Data [file qzaf109_supplementary_data.zip › Figure S8.jpg]

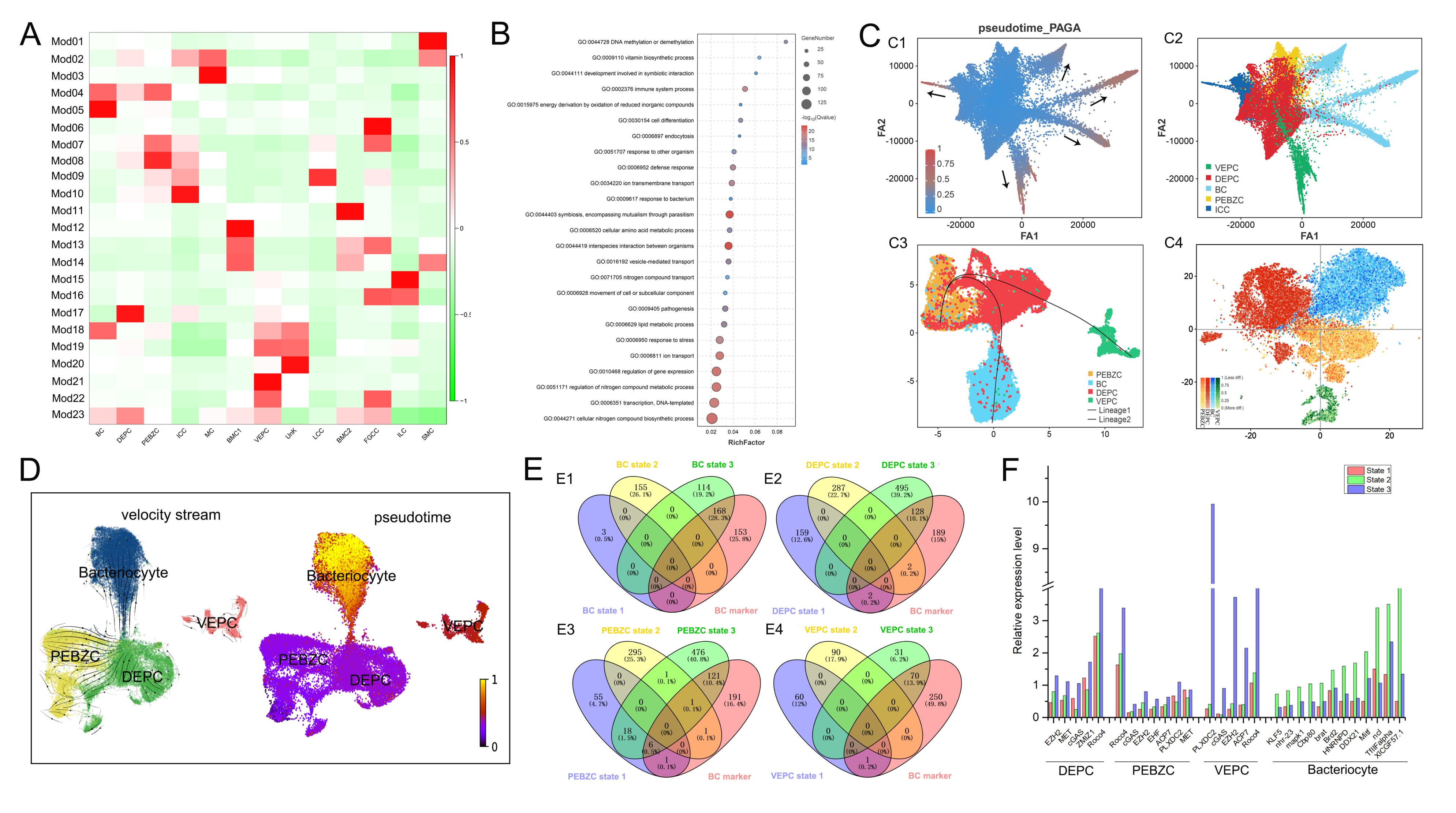

Supplement: qzaf109_Supplementary_Data [file qzaf109_supplementary_data.zip › Figure S9.jpg]
